# Is Follicle-Stimulating Hormone (FSH) Misnamed? Rethinking Its Role in Menopause as a Systemic Regulator of Bone, Brain, Metabolism, and Aging

**DOI:** 10.3390/jcm15176748

**Published:** 2026-08-31

**Authors:** Deanna Minich, Emily Hernandez, Caitlin Holmes, Douglas Lucas, Mona Fahoum, Wendy Warner, James Frame

**Affiliations:** 1 Symphony Natural Health Institute, West Valley City, UT 84119, USA; emily@emilyhernandez.co (E.H.); nutrition@caitlinholmes.com (C.H.); 2 Food & Spirit LLC, Port Orchard, WA 98366, USA; 3 Emily Hernandez Nutrition, Ventura, CA 93003, USA; 4 Caitlin Holmes Nutrition LLC, Santa Fe, NM 87505, USA; 5 The OsteoCollective LLC, Asheville, NC 28732, USA; doctorlucas@gmail.com; 6 Meridian Medicine, Seattle, WA 98133, USA; drmona@meridianmedseattle.com; 7 Wendy Warner, MD PC, Yardley, PA 19067, USA; dr@wendywarnermd.com; 8 Symphony Natural Health Holdings Inc., Craigmuir Chambers, Road Town, Tortola VG1110, British Virgin Islands; james.frame@naturalhi.com; 9 Natural Health International Pty Ltd., Sydney, NSW 2000, Australia

**Keywords:** perimenopause, postmenopause, biomarkers, osteoporosis, nutrition, botanicals, lifestyle, aging, longevity

## Abstract

Follicle-stimulating hormone (FSH) has long been recognized as a reproductive hormone in women and men. Based on research findings over the past decades, this classification may be incomplete and is increasingly being challenged. With this review, it is proposed that the name may be somewhat misleading. In addition to its presence in men without ovarian follicles, accumulating preclinical and clinical evidence suggests that FSH may serve as a marker of upstream endocrine changes across the stages of menopause. It may also participate in systemic processes that go beyond the classical hypothalamic–pituitary–gonadal axis. A less well-known finding is that FSH often rises before estrogen declines and is, to some extent, independent of estradiol. Moreover, changes in FSH levels through menopause coincide with considerable shifts in bone density, body composition, vascular function, neurobiology, and immune regulation. Based on what is known about FSH receptors, it is plausible that the systemic effects observed during menopause could result from extragonadal tissues’ receptivity to FSH. In this review, we present the traditional framework for FSH alongside recent findings that warrant re-evaluation of its function as a hormone, particularly in women as a modifiable marker of endocrine aging. We review clinical interventions for FSH, including laboratory biomarkers, hormone therapy, nutrition, botanicals, and lifestyle factors such as exercise and environmental exposures.

## 1. Introduction

Over the past two decades, perimenopause and menopause have emerged as prominent topics in medical and public discourse. This renewed emphasis followed the critical reassessment of the Women’s Health Initiative (WHI) findings, which contributed to a broader reexamination of the role of hormones in women’s health [1,2,3,4]. Moreover, awareness of perimenopause as a specific and distinct stage of reproductive aging has heightened. This groundswell of interest has led women, researchers, and clinicians to seek a new understanding of the changes that occur during this physiological transition.

Through this paradigm shift, it has been said that menopause is “having a moment” [5]. Numerous gaps in understanding this time of life have been identified. Some of these include the paucity of biomedical research on women, especially women of diverse races and ethnicities [6,7], and the gross inadequacy of medical–clinical training programs in menopause care [8]. Additionally, several topics have been questioned and more actively debated after long-held beliefs within women’s endocrinology, including the recent renaming of PCOS (polycystic ovarian syndrome) to PMOS (polyendocrine metabolic ovarian syndrome) [9], closer scrutiny of bioidentical hormones [10,11], extensive analyses of whether estrogen therapy increases the risk of breast cancer [12], and the incidence and use of individualized testosterone replacement [13], to name a few. These developments underscore a unique moment in history, marked by a collective reassessment of longstanding beliefs and clinical practices in women’s endocrinology.

While there has been a broader look at and reversal of several areas within women’s health, the majority of this renewed interest in the phases of menopause (perimenopause, menopause, and postmenopause) has focused on exogenous hormone replacement, especially estradiol, to the extent that transdermal estradiol patch shortages were reported in 2026 [14]. Truly, there is no mistaking the fact that the decline in the production of estrogen by the ovaries is one of the classic hallmarks of menopause and requires attention [15]. It drives many physiological, emotional, and mental shifts that occur during this transition, including vasomotor symptoms, bone loss, alterations in body composition, changes in mood and cognition, and increased cardiometabolic risk [16]. Due to the depth and breadth of this hormone’s impact, menopause research and clinical care have mostly focused on addressing estrogen deficiency, with careful re-evaluation of the potential for pharmacological replacement after the initial WHI findings [17]. A little more than twenty years after the WHI study was published, menopausal hormone therapy (MHT) now serves as a more mainstream strategy for some clinicians to address symptoms and support long-term personalized health concerns in appropriate patients during midlife and beyond [4,18].

The pendulum swing in the perception of estrogen, from the more cautionary aspects identified due to the WHI study to the contemporary, more curious mindset, continues to shape advancements in women’s health. It also highlights a shortcoming in endocrine medicine: the tendency to interpret complex physiological transitions through the lens of a single hormone (in this case, estrogen). Yet, this thinking is at odds with what has long been known: that endocrine physiology operates with greater complexity through interconnected signaling networks in which hormones interact dynamically via feedback loops, tissue-specific receptors, and cross-system communication [19]. Oversimplifying the endocrine system to focus solely on estrogen decline in perimenopausal women can lead to overlooking other endocrine changes, such as those affecting metabolic health, that may contribute to or further influence the menopausal transition [20].

This challenge is particularly relevant as the definition of the endocrine system itself continues to expand beyond the classical view of a linear, downstream network of glands [21,22]. The understanding of the endocrine system has now evolved to include extragonadal tissues and organs, such as adipose tissue [23], bone [24], skeletal muscle [25], heart [26], and the gastrointestinal tract [27], each of which participates in extensive hormonal crosstalk. This expansive systems biology perspective suggests that reproductive aging may involve coordinated adaptations across multiple endocrine tissues rather than changes in estrogen alone [22].

One of the most striking yet comparatively underappreciated endocrine changes in midlife that this review brings to the forefront, in the current, ever-changing milieu of debate and debunking within women’s health, is the marked rise in follicle-stimulating hormone (FSH) that occurs during the late reproductive phase [28,29]. Most clinicians would associate FSH solely with reproductive capacity; however, there is more to consider. As extrapolated from the Stage Reproductive Aging Workshop (STRAW)+10 framework [28], FSH increases dramatically across the menopausal transition, but that rise often occurs years before sustained declines in estradiol become apparent. Therefore, it could be argued that it is one of the largest hormonal shifts observed in female physiology.

Historically, this rise with the transition from the reproductive to menopausal stage of life has been interpreted primarily as a compensatory response to declining ovarian function and reduced negative feedback from the ovaries [29]. It is rarely given clinical attention after menopause. Moreover, the primary WHI intervention trial did not feature FSH as a biomarker [30]. Still, subsequent studies using stored specimens from WHI have assessed the role of FSH in postmenopausal obesity and breast cancer [31,32,33]. Thus, to date, FSH has mostly been viewed as a biomarker of ovarian function and menopausal status rather than as a hormone with greater physiological significance to the whole endocrine system.

Another point of consideration is the evidence accumulating for FSH’s actions beyond the ovary. Interestingly, receptors capable of responding to FSH, termed follicle-stimulating hormone receptors (FSHRs), have been identified in several cell types and tissues [34], including osteoclasts (but not osteoblasts) [35], adipocytes [36], as part of atherosclerotic plaques (includes several cell types such as certain endothelial cells, macrophages, and foam cells) [37], diverse tumor cells [34], monocytes [38], hepatocytes [39], and neuronal populations [40]. If FSH were solely a reproductive hormone, its effects would be limited to the gonads rather than being extragonadal.

In parallel with a discussion of these cell receptor activities, this review will provide a summary of experimental and clinical studies that associate FSH with the physiological responses seen through bone remodeling [41], energy metabolism [42,43], lipid regulation and vascular inflammation [44], and neuroendocrine function [45,46,47] and other processes that become particularly relevant during midlife, menopause, and aging. Along these lines, there is a suggestion that FSH may influence longevity through these activities [48,49]. Lizneva et al. [50] have proposed that it is now time to question whether FSH is a “true aging hormone”.

Finally, despite the absence of ovarian follicles, FSH has long been recognized as an important hormone in men, supporting Sertoli cell function and spermatogenesis [26]. This observation alone suggests that its name does not accurately reflect FSH’s physiological actions. Beyond its established reproductive roles, evidence of FSH activity in other tissues in men remains limited and, in some cases, inconsistent. Studies examining bone metabolism have demonstrated mixed findings [51,52,53,54]. Research on cardiometabolic and immune pathways remains preliminary and heterogeneous [55,56,57,58,59].

While many of these findings remain under active investigation, they prompt several important questions. Has FSH been understood and categorized too narrowly? Does assessment of FSH in clinical settings provide useful information earlier in the menopausal transition than is currently appreciated? Could modulation of FSH influence physiological outcomes beyond reproduction? And most fundamentally, does the term “follicle-stimulating hormone” adequately capture the full scope of its biological activity?

In this review, we examine the historical foundations of FSH biology, the evidence supporting its established reproductive functions, and the emerging preclinical and clinical literature suggesting more complex, intricate systemic actions. Additionally, we examine whether FSH should be viewed as more than a reproductive hormone and biomarker of ovarian aging. We consider the evidence that FSH may also be hinting at changes in endocrine function and whole-body physiology. This perspective is particularly relevant as the endocrine system is increasingly understood as an integrated network of tissues and signaling pathways rather than a linear series of glands, and menopause as a functional decline in the endocrine system instead of just the loss of estradiol.

## 2. The Traditional Model of FSH

### Historical Framework of FSH

**Figure 1:**
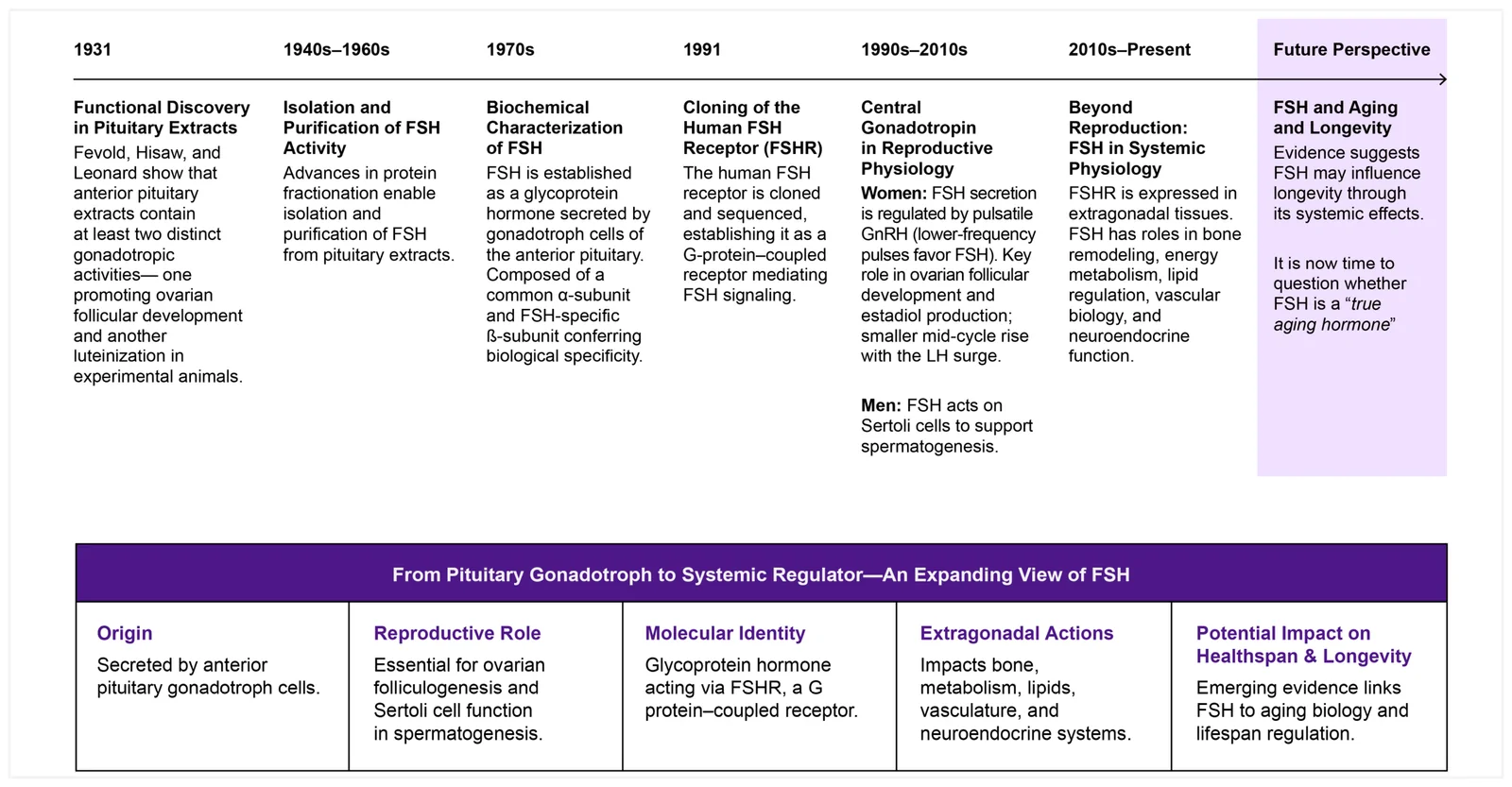
The Historical Framework of FSH [60,61,62,63,64,65,66,67,68,69,70].

It is worthwhile to start with the framework upon which the traditional definition and discovery of FSH were established before evaluating whether FSH warrants re-evaluation (Figure 1). For nearly a century, since its discovery, FSH has been viewed primarily as a reproductive hormone, based on early research. In the 1930s, Fevold, Hisaw, and Leonard at the University of Wisconsin conducted animal experiments and demonstrated that extracts of the anterior pituitary contained at least two distinct gonadotropic activities: one that induced ovarian follicular development and another that induced luteinization [60]. These activities were defined by their physiological effects, since the hormones had not yet been chemically isolated or structurally characterized. This research set the stage for one of the earliest functional distinctions between follicle-stimulating hormone (FSH) and luteinizing hormone (LH). It provided the foundation for their subsequent purification, biochemical characterization, and nomenclature in the following decades.

FSH activity was later isolated and purified from pituitary extracts from the 1940s through the 1960s using advancing protein fractionation techniques [61,62,63,64]. Further studies established FSH as a glycoprotein hormone secreted by gonadotroph cells of the anterior pituitary gland. It was composed of a common α-subunit shared with other glycoprotein hormones and a hormone-specific β-subunit that conferred biological specificity [65,66,67]. The cloning of the human FSHR in 1991 further advanced understanding of its biological actions and provided mechanistic insight into FSH-mediated signaling [68]. This milestone laid the groundwork for later findings of FSH in peripheral tissues.

Since its initial characterization, FSH has been widely recognized as a key gonadotropin in reproductive physiology in both sexes [71]. Within the hypothalamic–pituitary–gonadal (HPG) axis, pulsatile secretion of gonadotropin-releasing hormone (GnRH) from the hypothalamus stimulates gonadotroph cells in the anterior pituitary to release FSH and LH [72]. These hormones then act on the gonads to coordinate reproductive function through a tightly regulated endocrine feedback system.

In women, FSH secretion plays a key role in ovarian follicular development and estradiol production, with a smaller mid-cycle rise accompanying the LH surge [73]. It acts on granulosa cells in the ovary to promote follicular selection, growth, and maturation [74]. It further supports follicular development and ovarian steroidogenesis by facilitating the conversion of androgens to estradiol through stimulation of aromatase [75]. Therefore, FSH is closely related to estradiol, although not exclusively so. As the follicles mature, estradiol and inhibins participate in feedback regulation of the hypothalamus and pituitary gland [74]. In this manner, the menstrual cycle is maintained.

In men, FSH serves a parallel but distinct role in reproduction. Instead of stimulating ovarian follicles, FSH acts on Sertoli cells in the testes, where it supports spermatogenesis and helps maintain male fertility [54,71,72]. Together with testosterone, FSH regulates the development and maturation of spermatozoa and supports the integrity of the seminiferous tubules. Thus, despite its nomenclature, the physiological significance of FSH has never been restricted exclusively to ovarian follicles. Nevertheless, the appropriateness of its name has only recently begun to be questioned.

The interpretation of FSH within this reproductive framework has persisted for decades [76]. Clinically, elevated FSH concentrations have been used to assess ovarian reserves, characterize stages of reproductive aging, support the diagnosis of primary ovarian insufficiency, and confirm menopausal status [28,77]. In men, FSH measurements have been utilized in the evaluation of infertility and spermatogenic dysfunction [78]. Clinical guidelines and applications reinforced the prevailing assumption that the biological relevance of FSH resides primarily within the reproductive system [79].

This classical framework has served reproductive endocrinology well and remains foundational to our understanding of fertility and reproductive aging. Yet several observations have become increasingly difficult to reconcile with such a narrow interpretation [80]. If FSH functions solely as a gonadal hormone, why do its concentrations rise so dramatically during the menopausal transition, often years before sustained declines in estradiol become apparent? Why is FSH physiologically relevant in men despite the absence of ovarian follicles? And why have receptors capable of responding to FSH been identified in tissues with no obvious reproductive function?

These questions provide the basis for a growing re-examination of FSH biology and set the stage for a robust discussion regarding the physiological significance of this hormone beyond its historically recognized role in follicular development.

## 3. Evidence Challenging the Traditional Model

### 3.1. FSH Rises Before Estradiol Falls

One of the strongest indicators that FSH may signal more than fertility is that it begins to increase in a woman’s late reproductive years, usually before changes in estradiol levels are observed [28]. The rise in FSH during this period is relatively straightforward and well-established, beginning with the decline in ovarian follicles [29,81]. As the follicle pool diminishes, granulosa cells produce less inhibin B, a primary regulator of FSH secretion [29]. The reduction in inhibin B reduces negative feedback on the hypothalamic–pituitary axis, prompting the pituitary to increase FSH secretion to sustain ovarian function [29].

This physiological sequence helps explain why elevations in FSH frequently precede consistent or sustained declines in estradiol (and even progesterone). It is responding to inhibin B [81,82]. The dynamic among the follicles, inhibin B, and pituitary FSH release represents one of the earliest endocrine manifestations of ovarian aging. Based on this information, it could be posited that FSH elevations could serve as a clinical biomarker reflecting early dysregulation within the hypothalamic–pituitary–ovarian (HPO) axis [28,81,82,83,84].

FSH concentrations often increase despite relatively regular menstrual cycles and circulating estradiol levels within premenopausal ranges. This observation is supported by findings from the Study of Women’s Health Across the Nation (SWAN) and other longitudinal cohorts, which demonstrate that increasing FSH accompanies progression through the reproductive stages and is associated with approaching the final menstrual period, greater symptom burden, and changes in bone turnover [85,86,87,88].

**Figure 2:**
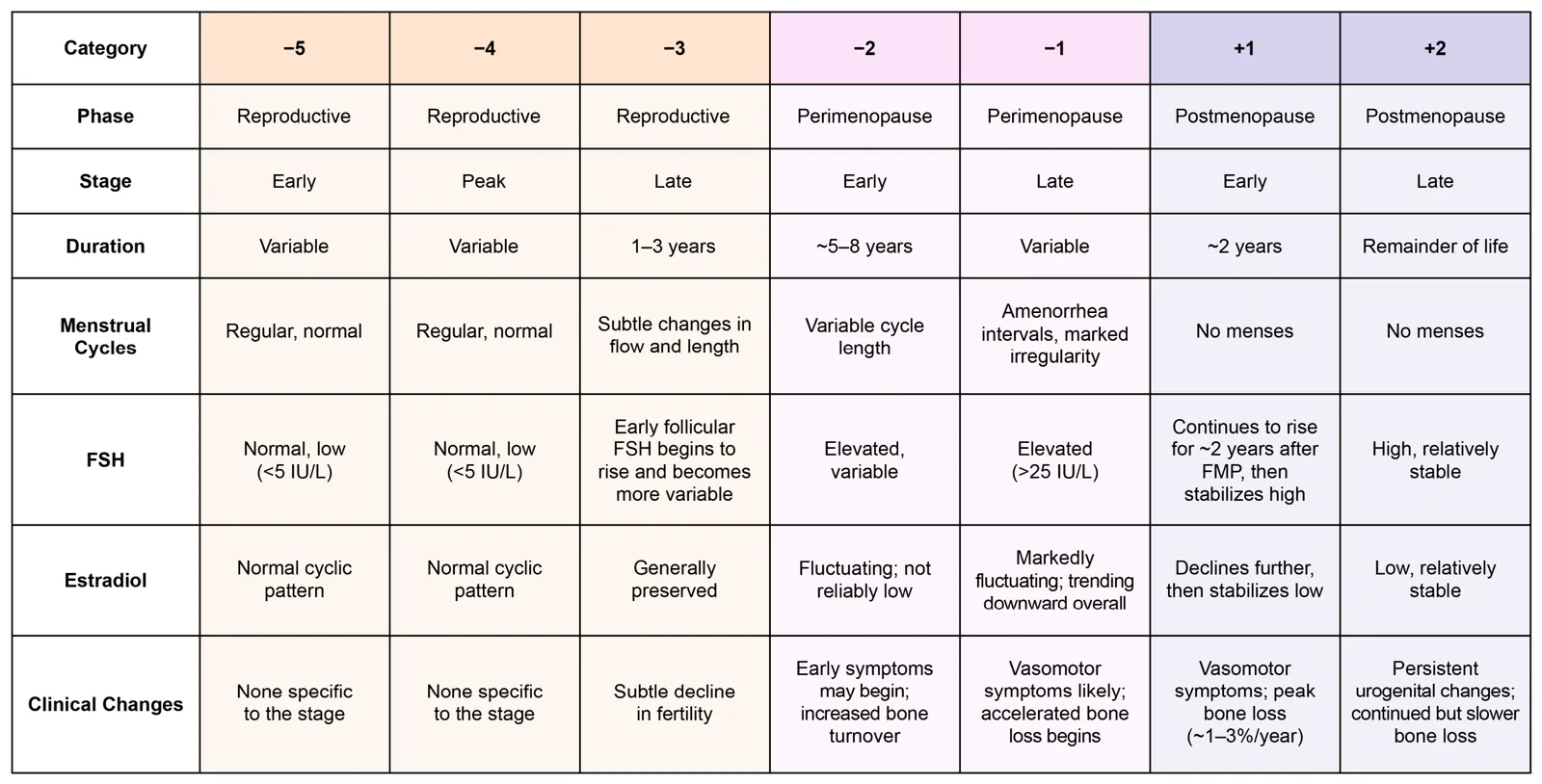
Reproductive Phases, FSH, Estradiol, and Clinical Changes. Adapted from [28,90].

The observation of an early rise in FSH before estradiol has been incorporated into the STRAW framework [89]. Within this system, reproductive aging comprises seven stages spanning the reproductive years, the menopausal transition, and postmenopause, including early, peak, and late reproductive phases; early and late menopausal transition; and early and late postmenopause (Figure 2). During the late reproductive stage (Stage −3), FSH levels begin to increase despite the presence of regular menstrual cycles [89]. Thus, the STRAW stages, as presented, recognize that endocrine changes begin well before the final menstrual period, with FSH typically rising in the late reproductive phase and into early perimenopause.

This hormone staging means that women may be exposed to elevated FSH for several years before substantial declines in estrogen occur, especially in those women experiencing early-onset perimenopause, who tend to have a longer transition to menopause. Whether this prolonged period of hormonal change has physiological consequences beyond reproduction remains an open question. Could rising FSH influence tissues outside the ovary long before menopause is reached?

From a menopausal trajectory perspective, it has been suggested that FSH levels begin to rise approximately 6 to 10 years before the final menstrual period [82,87,91,92]. However, with women experiencing early-onset perimenopause and a longer transition to menopause, there could be greater implications for exposure to elevated FSH for years before substantial declines in estrogen occur [93]. In other words, a prolonged period of FSH may have physiological consequences beyond reproduction, considering the emerging evidence for FSH receptors (FSHRs) in extragonadal tissues [94,95].

Even though FSH is one of the first hormones to signal the perimenopausal transition, its appearance is not always linear. There can be substantial variability both within and between individuals. Parry and Koch have referred to FSH as “Fluctuating Severely Hormone” [96]. Like most other hormones, during the early phase of perimenopause, FSH may fluctuate considerably from cycle to cycle, and even within the cycle itself. As will be discussed in more depth in the section on laboratory testing, a single FSH value has limited utility for assessing menopausal status. However, these fluctuations and the overall FSH pattern over time may provide important information about the endocrine system and even predict preclinical patterns in systems biology.

One might question the implications for the other gonadotropin, luteinizing hormone (LH). Although both FSH and LH increase during menopause, FSH has been more consistently implicated in extragonadal signaling across bone, adipose, vascular, and neural tissues, whereas LH remains more closely associated with reproductive steroidogenesis [97,98]. Nonetheless, emerging evidence from neuroendocrine–metabolic disorders such as polyendocrine metabolic ovarian syndrome (formerly referred to as polycystic ovarian syndrome or PCOS) [9] suggests that altered LH pulsatility may also reflect broader systemic regulatory functions extending beyond gonadal steroid production.

Overall, the takeaway is that FSH elevations can occur in the presence of normal or elevated estradiol [99], rise earlier and more variably during the menopausal transition [100], and are associated with outcomes such as reduced bone mineral density (BMD) [88], bone remodeling [41], and fracture risk [101]. These observations challenge the view of FSH as a direct and proportional indicator of estrogen deficiency and instead support its role as a partially independent endocrine signal within a broader neuroendocrine network [22].

One of the aims of this paper is to enhance clinical awareness so that FSH can be incorporated into clinical care earlier to assess and perhaps even treat accelerated endocrine aging earlier in life. Doing so may help to reduce or mitigate perimenopausal symptoms. If elevated FSH contributes independently to accelerated endocrine aging, it highlights the importance of supporting both ovarian and overall endocrine function throughout life. This perspective encourages maintaining hormonal balance and metabolic health for as long as possible and suggests that hormone therapy addresses only one aspect of the many physiological changes that occur during the menopausal transition.

It is also worth noting the dramatic difference in FSH levels between men and women. Women will reach a menopausal plateau nearly 14-fold higher than that observed in men. This stark difference may indicate that the effects of FSH in women may be of even greater importance [87,91,92].

Hence, this paper calls for a serious re-examination of FSH as a core feature in women’s health. If its role in reproductive aging, specifically in HPO dysregulation or an attempt at endocrine adaptation, can be better understood, then there is the potential for more integrative approaches to modulate this hormone at various times of life when there may be increased susceptibility to the development and onset of preclinical changes.

### 3.2. FSH Receptors Beyond the Ovary

In addition to the physiological hormonal dynamics observed over the years among inhibin B, FSH, and estradiol, emerging research on the cellular biology of FSH is helping put it in a new context. If FSH only had activity in the granulosa cells of the ovary [73], it would be plausible that its effects were limited to reproduction. However, a growing body of evidence, driven by advancements in molecular biology techniques, gene expression analyses, and immunohistochemistry, has challenged this view. Follicle-stimulating hormone receptors (FSHRs) have been identified in tissues outside the reproductive system [69,70,102]. Interestingly, these are the same tissues that undergo significant shifts during the menopausal transition, including potential involvement in bone remodeling, energy metabolism, lipid regulation, vascular biology, and neuroendocrine function [54,103,104].

While the preclinical evidence is discussed in greater detail in later sections, a brief overview is helpful here. Bone is one of the most extensively studied tissues to date. FSHRs have been identified on osteoclasts and osteoclast precursors, but not on osteoblasts [35]. Closely related to bone are immune-related cells. FSHR expression has been reported in mesenchymal stem cells and monocytes [38], suggesting a potential role in cellular differentiation and tissue remodeling. These findings might suggest that this mechanism could explain the increase in bone turnover and the acceleration of bone loss that occur during the menopausal transition.

Evidence has also accumulated for FSHR expression within adipose tissue [105,106]. Therefore, FSH may influence adipogenesis, lipid metabolism, energy utilization, and body composition more than originally realized, which would be particularly relevant given the significant changes in body composition and metabolism that occur during the menopausal transition [102]. Other peripheral tissues that express FSHR include vascular [37] and hepatic tissues [39], as well as neuronal populations within the central nervous system [40]. While the physiological significance of these findings remains under investigation, they may be involved in menopausal changes in cardiovascular health, lipid regulation, and even neuroendocrine shifts with aging.

More in-depth research is required into each of these areas to understand how receptor expression translates into clinically meaningful biological effects. Furthermore, it will be important to delineate how this receptivity may change during menopause, whether through FSH substrate availability, receptor density, tissue-specific activity, or signaling pathways. There may also be more than one receptor type, as seen with estrogen (ER-alpha and ER-beta), and these receptors change in activity with estrogen withdrawal. Still, as such, it is relevant that these tissues can receive the FSH signal.

Overall, the distribution of FSHRs beyond the ovaries represents an important physiological departure from the traditional framework of FSH biology. It brings forth the underlying question of whether the presence of these receptors contributes to some or all of the systemic changes observed during menopause. Furthermore, are there specific genetic variations in FSHRs that may be amplifying those signals in certain women more than others, such as in women with PCOS/PMOS [107,108]? Current efforts are underway to understand the FSHR, its genetic variability, and its potential as a therapeutic target in specific populations, ranging from reproduction to oncogenesis [109,110].

These questions about the FSHR and peripheral extragonadal activity of FSH provide the basis for linking FSH to bone health, body composition, metabolism, neuroendocrine function, vascular biology, immune regulation, and other physiological systems that undergo significant change during the menopausal transition [104,111,112,113,114]. These topics will be discussed in greater depth in subsequent sections, including preclinical and clinical evidence.

### 3.3. Evidence for FSH’s Role in Systems Biology Related to Menopause

Clinical and epidemiologic studies suggest that the rise in FSH during the menopausal transition occurs in tandem with physiological changes extending beyond the ovary. In the large SWAN multiethnic cohort of women aged 42 to 52 years, FSH increased during early perimenopause. At the same time, estradiol remained relatively stable, a pattern that was accompanied by changes in bone density [88,115,116]. Lumbar spine BMD declined across increasing FSH quartiles, with approximately 0.5% lower values observed in each successive quartile, independent of estradiol, testosterone, and sex hormone-binding globulin (SHBG) [88].

Similar findings have been reported in other cohorts. In a study of 2930 women aged 42 to 52 years, FSH was more closely related to menopausal stage than estradiol, testosterone, or dehydroepiandrosterone sulfate (DHEA-S) [117]. Clinical symptoms also appear to parallel the rise in FSH. In a cross-sectional study of 144 women, postmenopausal participants reported more frequent and severe menopausal symptoms than perimenopausal women, and higher serum FSH concentrations were associated with greater vasomotor symptom severity [118].

These physiological shifts may translate into psychological and neurobiological pathways. Spicer et al. [92] have suggested that FSH may represent a novel biomarker of mental health during the menopausal transition, with potential links to anxiety, depression, and broader neuroendocrine changes. Additional studies suggest potential roles for FSH in lipid metabolism and cholesterol regulation, as well as novel associations with other physiological processes like vascular endothelial health [102,119], although these findings remain under investigation. Overall, these data further align with the concept that estrogen deficiency alone may not fully account for the spectrum of changes observed during menopause and that FSH may represent an additional, and potentially underrecognized, endocrine signal of aging and/or adaptation [22].

While it may seem that FSH elevations are associated with adverse health outcomes during the menopausal transition, some evidence suggests context-dependent effects, with select clinical studies indicating that FSH may exert protective influences on metabolic pathways. Therefore, the role of FSH may need to be viewed as a complex endocrine signal rather than a uniformly pathogenic factor [33,44,120,121].

## 4. Systemic Effects of FSH

### 4.1. Bone

The skeleton has increasingly been recognized as an active endocrine organ that participates not only in structural support and mineral homeostasis, but also in systemic regulation of energy metabolism and hormonal signaling [122]. Bone produces hormones and signaling molecules that influence both local bone remodeling and broader cross-organ communication [123,124]. Bone-derived factors therefore affect multiple systems, including cardiovascular, reproductive, nervous, immune, and renal systems, as well as glucose and lipid metabolism and skeletal muscle function [123,124,125]. At the same time, the skeleton responds to the surrounding hormonal and metabolic milieu, driving continuous bone remodeling throughout life [124]. Bone remodeling becomes particularly relevant during the menopausal transition, when rising FSH and declining estradiol alter the balance between bone resorption and bone formation [35].

Clinical and epidemiologic data support a role for FSH in bone remodeling during the menopausal transition, showing that rising FSH levels coincide with alterations in bone density, despite relatively stable estradiol concentrations [88,115,116]. Clinical studies also consistently report an association between elevated FSH levels and lower BMD or increased bone turnover markers [41,126,127].

Chin [35] proposed that FSH may directly influence bone health independently of estrogen, in part by promoting increased bone resorption. This idea dates back to Steinberg et al. [128], who reported an inverse correlation between FSH and perimenopausal bone loss in 1989. Subsequent research on FSHR expression in bone cells has begun to clarify why this may occur. Whereas estrogen influences both osteoblast and osteoclast activity, FSH appears to act primarily through FSHRs expressed on osteoclast precursors and mature osteoclasts, which are bone-resorbing cells [35]. This osteoclast-specific mechanism is the most clearly established link between FSH and skeletal changes during menopause.

#### 4.1.1. Preclinical Research and Mechanisms

##### Cellular Mechanisms: FSH Signaling in Bone

FSHRs expressed on mesenchymal stem cells, osteoclast precursors, and osteoclasts mediate the effects of FSH on bone resorption [35]. FSHRs are not known to be expressed on osteoblasts [35]. Further, FSHR variants or isoforms expressed on osteoclasts differ from those in ovarian cells, though the functional relevance of this remains to be elucidated [38]. Mechanistic studies show that FSH promotes bone resorption by enhancing pathways that support osteoclast differentiation, activity, and survival [35].

In experimental models, FSH activates osteoclastogenic signaling pathways by enhancing phosphorylation of extracellular-signal-regulated kinase 1/2 (Erk1/2), protein kinase B (Akt), and inhibitor of kappa B alpha (IκBα), which collectively promote osteoclast differentiation [129]. FSH also increases receptor activator of NF-kB (RANK) expression, which is expressed on osteoclast precursor cells and is central to osteoclast differentiation [130]. In a human cell study, FSH stimulated RANK expression in a dose-dependent manner, with the greatest increase observed at FSH concentrations typical of perimenopause (50 IU/L), whereas higher concentrations typical of menopause (100 IU/L) had a lesser effect [130].

In addition, FSH upregulates cytokines, such as tumor necrosis factor-α (TNF-α), interleukin-6 (IL-6), and interleukin-1β (IL-1β), that promote osteoclast formation [126,131]. FSH may also facilitate actin ring formation in osteoclasts, which is essential for forming a resorption compartment and preventing osteoclast apoptosis [35,132,133]. Collectively, these findings support the role of FSH in directly enhancing osteoclastogenesis by coordinating intracellular signaling pathways and pro-inflammatory cytokine networks.

##### Animal Studies

Early animal research suggests that estrogen deficiency may not fully account for bone loss and that FSH may be an underrecognized regulator of bone metabolism. In animal models, surgical, genetic, or pharmacological manipulation of FSH signaling influences bone loss [129,134,135,136]. Compared with ovariectomized rats, rats that are both ovariectomized and hypophysectomized exhibit a blunted response to bone loss, which may reflect the absence of pituitary hormones, including FSH [135]. Further, a 2006 animal study showed that bone loss was absent in FSHβ- and FSHR-null mice, which have a complete loss of FSH signaling, despite severe estrogen deficiency [129]. In ovariectomized female rats, treatment with leuprorelin, a GnRH agonist that suppresses FSH, was associated with significantly decreased alveolar bone loss compared with non-treated ovariectomized female rats (*p* < 0.05) [134]. Later research using ovariectomized mice demonstrated that injection of FSH antibody attenuates bone loss by blocking osteoclast formation [136]. These results raise the possibility that FSH may contribute to bone loss independent of estrogen.

#### 4.1.2. Clinical Research

##### FSH and Bone Health in Menopause

The extragonadal role of FSH in bone metabolism has been extensively studied. In 1989, Steinberg et al. published a study describing bone loss in perimenopause and its inverse correlation with FSH levels [128]. Later research published in 1996 by Ebeling et al. [137] found a significant positive association between bone turnover markers and FSH concentration in menopausal women (*p* < 0.0001). Compared with premenopausal women, perimenopausal women exhibited higher FSH levels (*p* < 0.01) and a 20% higher urinary excretion of the bone resorption marker N-telopeptide (*p* < 0.05), yet similar estradiol and bone formation marker concentrations.

Nonetheless, perimenopausal bone loss has largely been attributed to estrogen decline. However, estradiol fluctuates during this time, and its trajectory is not uniform in all women [86]. SWAN data show that nearly half of the women assessed postmenopausally experienced significantly elevated estradiol levels before the final menstrual period [86]. Accelerated bone loss has been observed before consistent declines in estradiol, suggesting that additional factors may contribute to skeletal changes during perimenopause [81,138]. Apart from the proposed direct effect of FSH on bone metabolism, a cross-sectional study involving 669 postmenopausal women suggests that FSH may mediate approximately 70% of the association between estradiol and BMD (Sobel *p* < 0.001) [139]. The FSH trajectory during perimenopause and its stability in later life may additionally influence its effect on bone, in part due to changes in calcium regulation that coincide with bone loss during menopause [140].

Findings by Koh et al. [101] suggest that FSH is an independent marker of bone fracture risk in a case–cohort study involving both men and women. Using a model adjusted for age and sex, incident hip fracture risk increased 24% per sex-specific standard deviation increase in FSH [HR 1.24 (95% CI 1.04–1.48), *p* = 0.02]. This association remained significant after further adjustment for sex hormones, which attenuated the risk to 20% [HR 1.20 (95% CI 1.01–1.44), *p* = 0.04]. For women, in a model adjusted for age and sex hormones, each standard deviation increase in FSH (25.5 IU/L) was associated with a 22% higher risk of incident hip fracture, though it lost statistical significance [HR 1.22 (95% CI 0.92–1.61), *p* = 0.16]. In this context, FSH has been proposed as a potential contributor to bone loss independent of estradiol, although this remains an active area of research [126].

The effects of FSH on bone loss also appear to be site-specific. In a cross-sectional study of women aged 45–60 years, log-FSH is shown to be independently associated with lumbar spine BMD (*p* = 0.009), whereas log-estradiol was the only predictor at the femoral neck (*p* = 0.02) [141]. This may be due to differences in bone microarchitecture, as the lumbar spine is composed of trabecular bone, whereas the femoral neck is composed of cortical bone [142]. Compared with trabecular bone, cortical bone may undergo slower rates of bone loss during the menopausal transition, as its threshold for estrogen deficiency is lower [143]. As such, FSH is suggested to preferentially affect trabecular bone [144].

It is also important to recognize that much of the existing literature examining FSH and skeletal health relies primarily on dual-energy X-ray absorptiometry (DEXA)-derived BMD measurements. While BMD is a clinically valuable predictor of fracture risk, DEXA primarily assesses mineral content and does not completely capture other key determinants of bone strength, including collagen integrity, trabecular connectivity, cortical porosity, bone quality, and bone microarchitecture [145,146]. This is important because bone is a dynamic composite tissue composed not only of mineral but also of an organic matrix rich in type I collagen and non-collagenous proteins that contribute to flexibility, resilience, and structural integrity. Therefore, current findings linking FSH to “bone loss” should be interpreted within the context of mineralized tissue measurements rather than comprehensive assessments of skeletal architecture. Additional research using advanced imaging modalities and biomarkers of bone quality is needed to clarify whether FSH influences aspects of bone remodeling beyond changes in bone mineral density, including matrix composition, connective tissue integrity, and skeletal microarchitecture. Alternative imaging modalities may improve access to accurate bone health assessment and enable more frequent evaluations than DEXA. For example, Radiofrequency Echographic Multi Spectrometry (REMS) uses ultrasound technology to directly assess bone mineral density and bone microarchitecture at the femoral neck and lumbar spine [147]. A study involving postmenopausal women undergoing osteoporosis screening with REMS achieved sensitivity and specificity exceeding 90% at both the lumbar spine and femoral neck [148].

##### FSH and Bone Turnover Markers

Bone turnover markers have exhibited associations with FSH. However, FSH’s osteoclast-specific mechanism shapes the interpretation of downstream bone turnover markers. Whereas markers associated with osteoclast activity reflect bone resorption and potentially direct FSH signaling, markers associated with osteoblast activity reflect bone formation and broader bone turnover dynamics [149]. In this context, elevations in bone formation markers do not necessarily indicate net anabolic effects on bone, particularly when bone resorption remains disproportionately elevated.

Osteocalcin is one of the most abundant and well-studied non-collagenous proteins, or osteokines, produced by osteoblasts and contributes to bone mineralization and strength by aligning apatite crystals with collagen fibrils [124,150]. However, its measurement is complex: carboxylated osteocalcin has a high affinity for hydroxyapatite and is deposited into the bone, whereas undercarboxylated osteocalcin has a low affinity and is released into circulation. Although both forms can reflect bone turnover, uncarboxylated osteocalcin is also vitamin K-dependent and thus increases with low vitamin K status [151]. Total osteocalcin represents both forms, but its value as a marker of postmenopausal osteoporosis remains controversial [151].

Tanaka et al. (2020) demonstrated that undercarboxylated osteocalcin levels were highest in women aged 50–69 years compared to those aged 40–49 years and showed a significant negative correlation with quantitative ultrasound bone parameters [152]. Earlier research reported that undercarboxylated osteocalcin was negatively correlated with estradiol concentration (*p* < 0.0001) and positively correlated with FSH concentration (*p* < 0.0001) in pre-, peri-, and postmenopausal women [153]. Together, this likely reflects overall bone turnover dynamics, particularly since elevated undercarboxylated osteocalcin may indicate insufficient vitamin K status during periods of accelerated remodeling [152,153].

Like osteocalcin, procollagen type I N-terminal propeptide (P1NP), an indicator of osteoblastic bone formation and collagen synthesis, may increase as part of coupled bone remodeling dynamics secondary to heightened osteoclast activity [149,154,155]. Evidence suggests that rising FSH levels during the menopausal transition are also associated with increased P1NP. In a cohort of perimenopausal women, serum P1NP was modestly but significantly correlated with FSH (rs = 0.159, *p* = 0.009) [156]. Women aged 40–50 years with FSH concentrations ≥40 IU/L demonstrated significantly higher P1NP levels than those with lower FSH concentrations (51.33 ± 24.67 vs. 35.05 ± 18.11 ng/mL, *p* = 0.004). In multivariable regression analysis, FSH remained independently associated with P1NP, whereas age was not significantly associated with P1NP after adjustment [156]. However, the relatively small effect size and the coupled nature of bone remodeling suggest that these elevations in P1NP during the menopausal transition likely reflect increased skeletal turnover secondary to increased osteoclast activity.

In summary, FSH appears to predominantly influence bone by promoting osteoclast-mediated resorption, while osteocalcin and P1NP may reflect downstream remodeling responses to osteoblast activity. Overall, rising FSH appears to be linked to changes in bone formation markers because bone remodeling is coupled, despite the absence of FSHR expression on osteoblasts.

##### Perimenopause

Rising FSH during perimenopause reflects compensatory changes within the HPO axis, including decreased inhibin B, a peptide involved in FSH regulation [126]. Accordingly, FSH has the potential to serve as both an indirect indicator of declining ovarian function in perimenopause and a more reliable predictor of bone loss than estrogen [88,126].

In the large, ethnically diverse SWAN study of 2336 premenopausal and perimenopausal women aged 42 to 52 years, FSH, but not estradiol, was significantly and consistently associated with BMD in the femoral neck, hip, and lumbar spine (*p* = 0.0002 to 0.0011, depending on BMD site) [88]. Another study using SWAN data demonstrates the dual effects of estradiol and FSH on perimenopausal bone loss, showing that women with both lower estradiol and higher FSH were more likely to experience bone loss in the femoral neck and lumbar spine [157]. At the lumbar spine, each halving of estradiol was associated with a 10% (*p* < 0.0001) greater risk, and each doubling of FSH was associated with a 39% (*p* < 0.0001) greater risk of significant bone loss. At the femoral neck, each halving of estradiol was associated with a 12% (*p* = 0.01) greater risk, and each doubling of FSH was associated with a 27% (*p* < 0.001) greater risk of significant bone loss. Overall, FSH was the stronger predictor of bone loss, though both hormones were associated with skeletal changes during the menopausal transition.

A prospective observational study reported significant declines in lumbar spine BMD among pre- and perimenopausal women (mean age 48.2 ± 2.3 years) over 12 months, with greater declines in perimenopausal women. Perimenopausal women also had significantly higher FSH (*p* < 0.05), alkaline phosphatase (*p* < 0.05), calcium (*p* < 0.05), and osteocalcin (*p* < 0.05), as well as significantly lower estradiol (*p* < 0.05) [158].

The FSH trajectory across the menopausal transition may influence the rate of bone loss. In a 5-year longitudinal study of 56 women followed through the menopausal transition, women were categorized by high or low FSH trajectory [140]. Although both groups experienced declines in BMD, the high FSH group showed significantly greater decline rates for total body (*p* = 0.007) and femoral neck (*p* = 0.003) BMD. This suggests that both FSH levels and the rate and pattern at which FSH increases may help identify women at greater risk of accelerated bone loss during the menopausal transition.

Pan et al. assessed FSH levels and the severity of intervertebral disc degeneration (IVDD) across the menopausal transition in a cross-sectional study [159]. Results demonstrated significant associations between FSH and IVDD severity. In addition, lumbar IVDD worsened progressively across the menopausal transition as FSH increased. FSHR expression was also detected in patient samples of nucleus pulposus, the gel-like component of the intervertebral disc that provides the spine with strength and flexibility [159,160]. These findings suggest that FSH may have direct effects on intervertebral disc biology, extending its role beyond bone to other connective tissues of the spine.

##### Postmenopause

**Figure 3:**
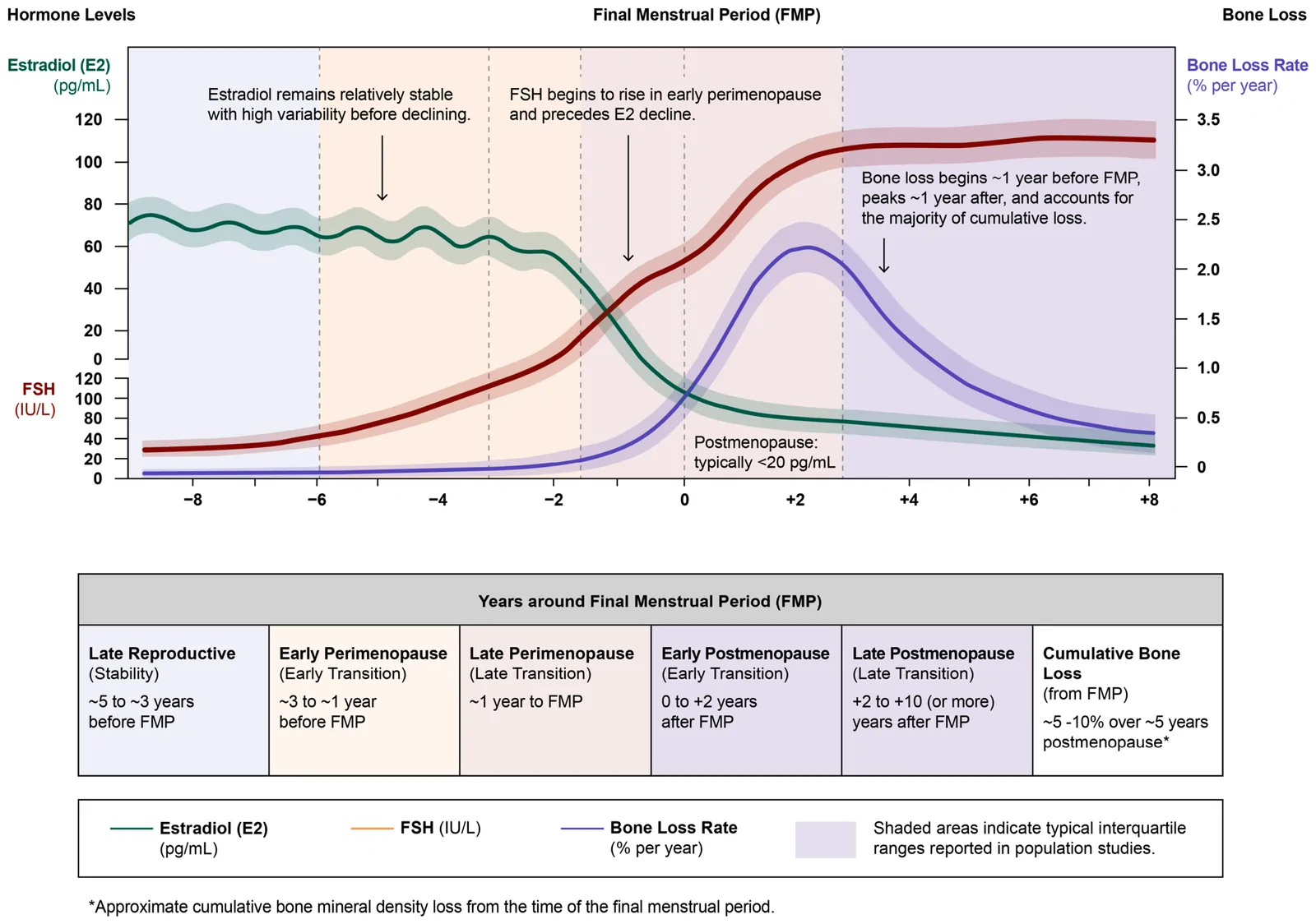
Estradiol, FSH, and Bone Loss Across the Menopausal Transition. Adapted from [28,91,157,162].

The relationship between FSH and BMD appears to vary across the menopause transition (Figure 3). SWAN data show that, despite a significant association between FSH and BMD change in perimenopause and early menopause, the association is lost in late postmenopause, defined as two years after the final menstrual period and thereafter [138]. A 3-year longitudinal study found that older postmenopausal women (mean age 80.9 ± 4.2 years at baseline) experienced bone loss over 3 years, but baseline FSH was not significantly associated with subsequent changes in BMD [161]. The authors speculate that more stable FSH levels in older age may attenuate the effects of FSH on bone loss compared with the rapid FSH changes in perimenopause.

The use of reproductive hormones, including anti-Müllerian hormone (AMH), estradiol, and FSH, as cost-effective tools for identifying women with increased risk of osteoporosis was examined in a prospective study of 312 postmenopausal women (>12 months since the final menstrual period) [163]. As BMD decreased, FSH increased significantly (*p* < 0.001), while AMH and estradiol decreased significantly (*p* < 0.001). All hormones were independent predictors of low BMD, though estradiol and AMH had slightly higher predictive ability than FSH. However, AMH concentrations are typically low or undetectable after menopause [164], and the clinical utility of AMH as a marker of bone health in postmenopausal women remains uncertain.

FSH is known to accelerate alveolar bone loss and cyclo-oxygenase-2 (COX-2) expression, which is implicated in inflammatory processes [165]. However, in one clinical trial of 60 postmenopausal women aged 45–57 years, researchers did not find a difference in FSH levels between women with and without chronic periodontitis [165]. Conversely, serum alkaline phosphatase levels were increased in the women with chronic periodontitis, suggesting a possible correlation with disease severity.

##### Genetic Variation in FSHR

Approximately 1800 single-nucleotide polymorphisms (SNPs) in the *FSHR* gene have been identified, some of which have been shown to influence FSH responsiveness [166]. Although research on *FSHR* gene mutations primarily focuses on their implications for reproductive outcomes, particularly in the context of in vitro fertilization (IVF) treatment, *FSHR* SNPs may also be associated with bone metabolism and osteoporosis risk [166,167].

Postmenopausal women possessing the AA (Asn680/Asn680) rs6166 *FSHR* genotype exhibited increased markers of bone turnover, decreased total body and femoral neck BMD, and lower stiffness index relative to the GG (Ser680/Ser680) rs6166 *FSHR* genotype, despite similar FSH and estradiol levels [167]. Other *FSHR* polymorphisms have also been associated with skeletal outcomes. For example, Cannarella et al. [168] found that postmenopausal Caucasian women aged 45–80 with the c.2039A>G *FSHR* GG genotype had the highest lumbar BMD z-score compared with those carrying the c.-29G>A AA promoter variant, showing the potential heterogeneity in genotype–phenotype relationships across populations and loci. In addition, Sopova et al. [166,169] found that the triple SNP combination (*FSHR* rs6166 AA, *TSHR* rs1991517 CC, and *ADRB2* rs1042713 AA) may have synergistic or interactive effects on bone metabolism through shared signaling pathways.

It may be worth mentioning that although interactions between or combined effects of SNPs in the *FSHR* and vitamin D receptor (VDR) genes have not yet been examined, alterations in FSH and vitamin D signaling pathways could collectively influence bone remodeling. Nonetheless, future research may identify genetic risk patterns involving the *FSHR* and *VDR* genes that affect skeletal health in peri- and postmenopausal women.

##### Intervention Studies

Hormone replacement therapy (HRT) [170] delivered orally or transdermally has been shown to increase serum estradiol levels and decrease serum FSH levels [171]. It is widely regarded as an effective measure in mitigating bone loss in menopausal women, especially when initiated within the first 10 years of menopause [172]. These skeletal benefits are attributed to restoration of estrogen signaling, which plays a central role in maintaining bone homeostasis.

In 1996, Ebeling et al. [137] reported lower bone resorption markers in postmenopausal women using HRT than in non-users, along with lower FSH and higher estradiol. In a retrospective study of 125 women taking HRT, Kawai et al. [173] found that lumbar spine BMD changes were inversely associated with FSH and positively associated with estradiol, with most changes occurring in the first 12 months.

An intervention study using endocrine suppression and stimulation tests to manipulate estradiol and FSH levels in premenopausal women undergoing IVF failed to demonstrate an effect of FSH on markers of bone turnover [174]. In postmenopausal women (ages 67.4 ± 1.2 years), treatment with a GnRH agonist reduced FSH levels into the premenopausal range [175]. However, they exhibited elevated serum bone resorption markers, C-terminal telopeptide of type I collagen (CTX) and tartrate-resistant acid phosphatase 5b (TRAP5b), suggesting that suppression of FSH alone does not necessarily reduce bone resorption in this context but may indicate broader endocrine changes instead of a direct therapeutic target.

Gera et al. [176] speculated that studies using GnRH modulators would reflect not only changes in FSH but also concurrent changes in GnRH and LH, potentially confounding results. To selectively inhibit FSH action, an FSH-blocking antibody, MS-Hu6, was developed; however, it has been evaluated only in an animal model [176]. In this model, ovariectomized mice injected with Ms-Hu6 for 8 weeks showed increased total body and femoral BMD. The authors suggest that MS-Hu6 blocks FSH without affecting estrogen or LH levels, likely because abundant FSHRs in the ovary continue to respond to bioactive FSH, hence maintaining ovarian responsiveness to lower FSH levels.

##### Summary

Of all the systems explored in relation to FSH in this review, bone makes the most compelling case for FSH’s physiological actions beyond reproduction. Of course, estrogen deficiency remains central to understanding menopausal bone loss, but rising FSH levels also appear to contribute to skeletal remodeling during this transition, particularly through its influence on osteoclasts. The other salient features are the timing of bone loss and hormonal changes. Bone loss often begins during perimenopause, when FSH is rising rapidly, and estradiol may still be relatively preserved. What remains unresolved is whether FSH acts independently, synergistically, or as part of a larger endocrine adaptation. Still, the skeletal literature challenges the long-held assumption that FSH is relevant only to ovarian function.

### 4.2. Body Composition: Adipose Tissue and Skeletal Muscle

#### 4.2.1. Adipose Tissue

Once regarded primarily as an energy storage depot, adipose tissue is now recognized as an active endocrine organ that secretes adipokines, inflammatory mediators, and hormones involved in appetite regulation and energy balance [177]. Rather than functioning as a single uniform tissue, it consists of several distinct depots that vary in location, color, cellular composition, and metabolic function. These include white adipose tissue, which primarily supports energy storage and endocrine signaling; brown adipose tissue, which drives thermogenesis and is relatively more mitochondrially rich; and beige adipose tissue, which exhibits some degree of thermogenic capacity [177,178]. Additional phenotypes, such as pink and yellow adipose tissue, further reflect specific roles in processes such as lactation and bone-associated metabolism, respectively [178]. These depots help form a coordinated network regulating energy balance, bone remodeling, metabolic signaling, and systemic physiology [178,179].

Some evidence suggests that adipose tissue may also respond directly to FSH [36], although findings are mixed [180]. Functional FSHRs have been identified in both human and murine adipocytes, where receptor activation promotes lipid accumulation and changes in adipokine secretion, including leptin and adiponectin, two key regulators of metabolic homeostasis [36]. Thus, through this receptivity in adipocytes, FSH influences their functions.

Adipokines released from adipose tissue act throughout the body, affecting the brain, liver, skeletal muscle, heart, and bone [177,181]. As a result, changes in adipocyte function have implications well beyond fat tissue itself. Theoretically, if FSH is impacting adipocyte biology, its effects could extend to other functions such as appetite regulation, insulin sensitivity, inflammation, and whole-body energy balance during the menopausal transition [156,181,182].

#### 4.2.2. Skeletal Muscle

Skeletal muscle is also now recognized as an endocrine organ that produces myokines, signaling proteins that communicate with adipose tissue, the liver, bone, and the central nervous system (CNS) to help regulate metabolism and energy homeostasis [183]. The relationship between higher FSH and lower lean mass in midlife women may reflect not just the impact of FSH on muscle itself, but also changes in the communication network among muscle, adipose tissue, and other endocrine organs.

#### 4.2.3. Preclinical Research, Mechanisms, and Clinical Translation

##### Cellular Mechanisms: FSH Signaling in Adipocytes

At the cellular level, FSH signaling in adipocytes appears to follow a Gαi-coupled pathway that increases intracellular calcium while lowering cyclic AMP (cAMP) [36]. This shift in signaling favors lipid storage over energy expenditure. As cAMP levels decline, thermogenic pathways become less active, including reduced expression of uncoupling protein-1 (UCP1), a mitochondrial protein that plays a central role in heat production by brown adipose tissue [106,184]. FSH has also been shown to increase the expression of several genes involved in lipid metabolism, including lipoprotein lipase, fatty acid synthase, and peroxisome proliferator-activated receptor-γ, changes that favor adipocyte differentiation and lipid accumulation [105,106].

##### Animal Studies: Effects on Adiposity and Energy Expenditure

Early experimental studies support a role for FSH in adipose biology and energy metabolism. In an avian model, FSH administration increased abdominal fat mass and upregulated FSHR expression in adipose tissue within hours [105]. In ovariectomized mice, blocking FSH signaling with antibodies directed against the FSHβ subunit reduced total body fat mass (*p* ≤ 0.01) [106]. The treatment also promoted the conversion of white adipose tissue toward a beige phenotype, increased mitochondrial density, and upregulated thermogenic genes, including *UCP1*, in both white and brown adipose tissue [106,112].

Similar findings have been reported in preclinical models, including diet-induced obesity, ovariectomy, aging, and FSHR haploinsufficiency [36,106,185,186]. Across these studies, inhibition of FSH signaling was associated with greater energy expenditure, higher oxygen consumption, increased thermogenic activity, and improvements in lipid metabolism [36,106,185,186]. The findings are not entirely consistent across species, however. In ovariectomized rats, FSH administration did not significantly affect body weight, body composition, food intake, or energy expenditure, despite measurable changes in lipogenic pathways and other aspects of metabolic function [170]. These differences highlight the complexity of FSH biology and suggest that its metabolic effects depend on factors such as species, experimental model, and physiological context.

#### 4.2.4. Clinical and Translational Evidence

Human studies have linked FSH with changes in adipose tissue, skeletal muscle, hepatic lipid metabolism, and cardiometabolic health. Most of this evidence comes from observational studies, so it is not yet possible to determine whether FSH contributes directly to these changes or simply reflects the endocrine changes that accompany the menopausal transition.

##### FSH and Body Composition Remodeling

FSH rises substantially with age, increasing approximately tenfold in postmenopausal women and threefold in men over 60 years of age [36]. These increases occur alongside common age-related changes in body composition, including an accumulation of visceral fat and a reduction in skeletal muscle mass. These indications may suggest some degree of association between age-related elevated FSH and metabolic dysregulation [187]. Although for men, testosterone levels (free and total), rather than FSH, may be a stronger driver of body weight changes [92,188].

FSH may also contribute to the loss of skeletal muscle mass during the menopausal transition [189]. In a cross-sectional study of women aged 30–70 years, appendicular lean mass index was inversely correlated with FSH (*p* = 0.003), but not with estradiol or testosterone in peri- and postmenopausal women [189]. Contrary to established thinking, these findings suggest that higher FSH levels, rather than declining estrogen alone, may contribute to unfavorable shifts in body composition, including reductions in lean mass. This insight could shed new light on the development of therapeutic strategies for women going through the menopausal transition, where the prevalence of sarcopenia is markedly higher in later perimenopausal (30%) and postmenopausal (27–32%) women compared to early-stage perimenopausal women (3%) [189].

In a cohort of 667 postmenopausal women from the WHI Buffalo OsteoPerio Ancillary Study [139], associations between serum FSH, estradiol, and DEXA-derived adiposity measures were evaluated using both cross-sectional and 5-year longitudinal analyses. In cross-sectional analyses adjusted for age, years since menopause, smoking, and hormone therapy use, FSH was inversely associated with all adiposity measures, including percent body fat, total fat mass, and regional fat depots. These associations were independent of circulating estradiol. In longitudinal analyses, among women who discontinued hormone therapy during follow-up (*n* = 242), FSH increased (+33.9 IU/L) while estradiol decreased (−44.3 pg/mL), alongside increases in all adiposity measures. In adjusted models, increases in FSH were associated with increases in percent total body fat, total fat mass, and subcutaneous adipose tissue (SAT). The longitudinal findings reflect change-on-change relationships, indicating that rising FSH over time tracks with increasing adiposity, even though higher FSH is associated with lower adiposity in cross-sectional analyses.

Although midlife weight gain has often been attributed to aging independent of menopausal status, the preferential accumulation of visceral adipose tissue appears more closely linked to the menopausal transition itself [102,190,191]. Observational studies report associations between elevated FSH concentrations and increased waist circumference, waist-to-hip ratio, and visceral fat volume, suggesting that rising FSH may contribute to central adiposity [102,192]. However, rather than acting as an isolated driver of adiposity changes, FSH likely interacts with lifestyle, demographic, and metabolic factors.

Conversely, some longitudinal studies have reported lower circulating FSH levels in postmenopausal women with higher BMI, potentially reflecting negative feedback from peripheral estrogen production via aromatization in adipose tissue [86,193]. Along similar lines, in a longitudinal analysis of postmenopausal women from the WHI Buffalo OsteoPerio study [33], baseline FSH was evaluated in relation to adiposity measures up to 17 years of follow-up. Higher baseline FSH was consistently associated with lower adiposity across all measures over time. Specifically, each 1 IU/L increase in baseline FSH was associated with 0.54 cm^2^ lower VAT, 1.11 cm^2^ lower SAT, 0.05 kg/m^2^ lower BMI, 0.04% lower body fat percentage, and 85 g lower total fat mass. Despite these inverse associations, adiposity increased over time in the cohort. VAT increased by 5.84 cm^2^ over 5 years and 20.5 cm^2^ over 17 years, both statistically significant. SAT also increased significantly over 17 years by approximately 12.1 cm^2^. These findings indicate that although higher baseline FSH is associated with lower adiposity over time, overall adiposity continues to rise with age during the postmenopausal period.

##### Summary

Changes in body composition during the menopausal transition have long been attributed to declining estrogen. At the same time, our understanding of adipose tissue and skeletal muscle has changed considerably. Rather than serving only as sites of fat storage and movement, these tissues are now recognized as active endocrine organs that communicate with the brain, liver, bone, and immune system through a variety of signaling molecules.

This shift in thinking has naturally led to questions about where FSH fits into the picture. Experimental studies show that adipocytes express functional FSHRs and respond to FSH through pathways involved in lipid storage, thermogenesis, and energy expenditure. Clinical studies have also linked rising FSH with changes in visceral fat, lean mass, and sarcopenia during the menopausal transition. The findings are not always consistent, which is perhaps expected given that body composition is influenced by many factors, including aging, estrogen, adiposity, physical activity, and overall metabolic health. Even so, FSH continues to appear in studies of these changes. Whether it is directly involved or simply reflects the hormonal shifts occurring during midlife remains uncertain.

### 4.3. Metabolism

The relationship between FSH and metabolism extends beyond body composition to include glucose and lipid regulation. In a cross-sectional study of 2121 perimenopausal women, higher FSH levels were associated with lower triglyceride concentrations (*p* < 0.01), and this relationship remained significant after adjustment for multiple cardiometabolic factors (*p* = 0.002) [194]. FSH was also inversely associated with fasting glucose, although this relationship was no longer significant after adjustment for BMI, suggesting that adiposity may account for at least part of the association.

Similar observations have been reported in postmenopausal women. In one cross-sectional study, FSH was independently associated with adiponectin (*p* < 0.001) and the leptin-to-adiponectin ratio (*p* = 0.008), even after adjustment for BMI, age, and years since menopause [195]. FSH was also related to BMI and Homeostatic Model Assessment of Insulin Resistance (HOMA-IR), supporting a connection between FSH, adipose tissue, and insulin sensitivity.

#### 4.3.1. FSH and Mitochondria

In an experimental study of human granulosa cells collected during IVF procedures from women aged 20 to 35 years, FSH increased reactive oxygen species (ROS) production at both physiological and supraphysiological concentrations, with the greatest increase occurring within mitochondria [196]. Higher FSH concentrations also increased mitochondrial respiration and ATP production. Despite the increase in ROS, there was little evidence of oxidative damage, suggesting that these reactive oxygen species may function as part of normal mitochondrial signaling rather than reflecting oxidative stress.

Menopause presents a very different hormonal environment. Instead of the cyclical fluctuations seen during the reproductive years, FSH remains chronically elevated [86]. Whether prolonged exposure to FSH has similar effects in non-reproductive tissues is not yet known. However, adipose tissue and bone both express FSHRs [35,36], presenting the possibility that mitochondrial responses could differ outside the ovary. Supporting this idea, animal studies have shown that blocking FSH increases mitochondrial density and thermogenic activity in adipose tissue [106]. At the same time, estradiol is declining, reducing the expression of antioxidant enzymes and signaling pathways that support mitochondrial function and biogenesis [197,198]. These observations suggest that the effects of FSH on mitochondrial function are likely influenced by the tissue under study, the surrounding hormonal environment, and the duration of FSH exposure.

#### 4.3.2. FSH and Hepatic–Lipid Axis

FSH has also been implicated in systemic lipid metabolism through associations with circulating lipids. In a study of 400 postmenopausal women, higher circulating FSH concentrations (≥78.3 IU/L) were associated with significantly higher low-density lipoprotein cholesterol (LDL-C) levels compared with lower FSH levels (40–78.3 IU/L) (*p* < 0.01) [39]. A reduction in FSH following hormone replacement therapy was accompanied by improvements in LDL-C.

However, findings across populations are not uniform. In a cross-sectional study of 411 postmenopausal women, higher FSH levels were independently associated with lower LDL-C after adjustment for age, estradiol, BMI, and other cardiometabolic factors (OR 0.185, 95% CI 0.051–0.669), while lower FSH levels were associated with increased odds of dyslipidemia [199]. These divergent findings suggest that the relationship between FSH and lipid metabolism may be context-dependent, influenced by adiposity, metabolic status, and stage of endocrine aging.

Another factor to consider relative to this discussion of lipids is that because cholesterol is a precursor for steroid hormone synthesis, these clinical observations may suggest that FSH-associated lipid alterations could indirectly affect endocrine regulation during aging [199,200], although this remains speculative.

#### 4.3.3. Summary

The relationship between FSH and metabolism is more complex than that observed for bone. Studies have linked FSH with triglycerides, insulin resistance, adipokine signaling, body composition, and lipid metabolism, but the findings are not always consistent. This variability is likely due to multiple factors influencing metabolic health, including adiposity, menopausal stage, hormonal environment, environmental toxicants, and lifestyle.

Metabolism is regulated through constant communication among adipose tissue, skeletal muscle, the liver, endocrine organs, and the central nervous system. The reported associations between FSH and adiponectin, leptin, HOMA-IR, lipid metabolism, and mitochondrial function suggest that FSH may be part of this network, but its precise role remains unclear.

As women move through the menopausal transition and FSH levels rise, changes in body composition, energy metabolism, lipid metabolism, and insulin sensitivity often occur simultaneously. Whether FSH contributes to these changes or simply reflects the broader endocrine adaptations of midlife remains an open question.

### 4.4. Cardiovascular System

Some evidence suggests that FSH may be involved in cardiometabolic health through its effects on lipid metabolism and inflammatory signaling [36,105,106]. Experimental studies indicate that FSH may act on metabolic tissues that express FSHRs, influencing pathways related to lipid metabolism and inflammation. However, evidence for functional FSH signaling in the human liver or immune system remains limited, and any link between FSH and atherosclerosis remains largely hypothetical [44].

The clinical evidence is also mixed. Although studies have reported associations between FSH and various cardiometabolic outcomes, the findings are not always consistent, and a direct role for FSH in metabolic syndrome or cardiometabolic disease has not been established. This is perhaps not surprising given the many factors that influence cardiometabolic health, including estradiol, adiposity, inflammation, aging, and lifestyle. Rather than acting on its own, FSH is likely one of several hormonal signals that change during the menopausal transition and may contribute to the metabolic changes observed during this time.

#### 4.4.1. FSH and Vascular Remodeling Across the Menopausal Transition

Studies examining FSH and subclinical atherosclerosis have not all reached the same conclusion. Much of the difference appears to depend on where women are in the menopausal transition. In SWAN, 856 women were followed for an average of 9.6 years to examine changes in estradiol and FSH in relation to vascular health [85]. Women with lower estradiol trajectories had greater progression of carotid intima-media thickness (cIMT) and a higher prevalence of carotid plaque after menopause. Steeper increases in FSH were also associated with less favorable vascular profiles, even after accounting for estradiol and traditional cardiovascular risk factors. These results suggest that changes in FSH over time may provide different information than a single FSH measurement.

Different findings were observed for women beyond menopause. In a cross-sectional study of 587 postmenopausal women, cIMT decreased across increasing FSH quartiles (0.94, 0.91, 0.87, and 0.85 mm; *p* for trend < 0.001), and this relationship remained after adjustment for estradiol and other cardiometabolic risk factors [120]. Estradiol was not associated with cIMT in that study.

Similar findings have not been reported in all populations. In a study of 227 climacteric women aged 40–59 years, higher FSH concentrations were associated with greater odds of increased cIMT (OR 1.021, 95% CI 1.009–1.033), independent of traditional cardiovascular risk factors [201].

Looking across these studies, there is no single relationship between FSH and vascular health. The associations appear to differ during the menopausal transition compared with later postmenopause. That may reflect differences in the underlying hormonal environment, changes in body composition and metabolism, or other factors that influence vascular biology. At this point, it seems more appropriate to view FSH as one part of the endocrine changes occurring during midlife than as an independent vascular risk factor.

#### 4.4.2. Summary

The cardiometabolic and vascular findings are more difficult to interpret than the bone findings. Associations between FSH and lipids, insulin resistance, inflammation, and vascular remodeling have been reported, but the results are not always consistent. That is perhaps expected given the many factors that influence cardiometabolic health during midlife, including aging, adiposity, estradiol, inflammation, and lifestyle.

### 4.5. Brain

Interest in FSH has also expanded beyond bone and metabolism to include the brain. Alzheimer’s disease (AD) disproportionately affects women, who account for approximately two-thirds of cases worldwide, and the risk of cognitive decline increases during the menopausal transition [46,113,202]. Much of this has been attributed to declining estrogen. However, more than two decades ago, reviews of the WHI Memory Study and other epidemiological data raised the possibility that rising gonadotropins might also contribute to neurological aging and AD risk [203,204]. More recent studies have continued to explore this idea, with particular attention to FSH as a potential contributor to age-related changes in the brain [202,205].

#### 4.5.1. Preclinical Research and Mechanisms

##### Cellular Mechanisms: FSH Signaling in the Brain

The central nervous system integrates signals from multiple endocrine organs while also regulating hormone production through the hypothalamic–pituitary axis [206]. As women move through the menopausal transition, changes in circulating hormones are likely to influence this communication, although the specific contribution of FSH has not been well studied [50].

Recent studies have identified FSHRs in several brain regions involved in learning, memory, and metabolic regulation, including the hippocampus and cerebral cortex [113,187,205]. In mouse models, the FSHR has been detected on both neurons and glial cells, suggesting that FSH may act directly within the brain rather than only through peripheral tissues [205].

Experimental studies also suggest that FSH influences pathways involved in neuroinflammation, synaptic function, and cellular metabolism [187,207]. In one mouse study, FSH increased the expression of the pro-inflammatory cytokines, TNF-α and IL-1β, in a dose-dependent manner, and these changes were accompanied by depression-like behaviors and reduced synaptic plasticity (*p* < 0.01) [207]. Whether similar effects occur in humans is not yet known.

Cholesterol metabolism provides another possible connection. Cholesterol is essential for synapse formation, membrane integrity, and amyloid precursor protein processing in the brain [208,209]. FSH has been shown to regulate cholesterol biosynthesis in peripheral tissues, including the liver [200], but whether it has a similar role in the brain has not been established. Because disturbances in brain lipid metabolism are closely linked with Alzheimer’s disease, this remains an area of interest [46,205].

The brain is also part of a larger endocrine system that includes bone, adipose tissue, and other metabolically active organs. Bone-derived osteocalcin has been linked to neurotransmitter synthesis and cognitive function [210]. Although there is no evidence that FSH directly regulates osteocalcin, changes in bone remodeling during the menopausal transition could indirectly influence signaling between bone and the brain. Adipose tissue also communicates with the brain through hormones and cytokines such as leptin, helping regulate appetite, energy balance, and hypothalamic function [181]. These observations suggest that changes in FSH may extend beyond individual tissues and reflect broader endocrine adaptations during reproductive aging [50,112].

##### Animal Studies

Preclinical evidence supporting a potential direct role of FSH in neurodegeneration comes from animal models of AD. In a murine model of AD, FSH has been shown to act directly on hippocampal and cortical neurons, accelerating amyloid-β and tau deposition and contributing to cognitive decline [205]. Blocking FSH signaling using neutralizing antibodies altered the AD-like phenotype and improved cognitive performance. These effects were mediated through the inhibition of the neuronal C/EBP-β-δ-secretase pathway, providing evidence for a potential role of FSH in neurodegenerative signaling [205].

A preclinical study involving male mice injected with recombinant human FSH supports these findings, indicating that FSH signaling may impair synaptic plasticity, induce neuroinflammation, and contribute to abnormal glutamate and gamma-aminobutyric acid (GABA) signaling [207]. Additional cell research by Huang et al. [207] demonstrates that FSH-related neuroinflammation may be mediated by activation of the Erk pathway, which is also implicated in FSH’s role in bone loss [129]. Collectively, these data provide mechanistic support for a contributory role of FSH in neurobiological processes.

#### 4.5.2. Clinical and Epidemiological Evidence

The relationship between FSH and cognitive decline remains an area of active investigation in human studies. The increase in FSH levels during the menopausal transition coincides with changes in cognition, mood, and brain structure [36]. Clinical research indicates that women are at higher risk for AD than men, with risk increasing after menopause [113,202].

While estrogen deficiency has long been the primary focus in studies of brain health during menopause, there is growing interest in whether rising FSH may also contribute to cognitive decline and neurodegenerative disease [211]. Perhaps surprisingly, this question has been around for many years. Earlier investigators proposed that gonadotropins, in addition to estrogen loss, might influence brain aging [203]. The WHI Memory Study renewed interest in this idea after reporting an increased risk of dementia with certain hormone therapy regimens, leading researchers to look more broadly at the hormonal changes that occur during menopause [203,204,212]. More recent studies have focused specifically on FSH and its possible role in brain aging and Alzheimer’s disease.

For example, cohort studies report that higher FSH levels are associated with worse cognitive performance scores or increased risk of cognitive impairment [113,202]. In addition, higher FSH levels in postmenopausal women were associated with greater white matter hyperintensity volume, a measure of cerebral small-vessel damage [213]. This relationship persisted after adjustment for traditional cardiovascular disease risk factors, although it was attenuated after accounting for sleep and vasomotor symptoms, suggesting that menopausal disruption of sleep may be partially responsible. Findings remain heterogeneous across populations. These inconsistencies likely reflect the complex interplay between FSH, menopausal stage, estrogen levels, metabolic health, and vascular risk factors during aging [113,202].

In a translational neuroimaging study of 191 midlife women (40–65 years of age) at risk for Alzheimer’s disease, higher circulating FSH levels were associated with greater β-amyloid deposition and lower gray matter volume in frontal brain regions [214]. These relationships were strongest in postmenopausal women but were already detectable during perimenopause. The associations persisted after adjustment for age, estradiol, APOE-4 status, hormone therapy use, and other clinical variables, suggesting that they were not explained solely by estrogen. FSH was more consistently associated with these brain changes than LH and was also marginally associated with lower hippocampal volume. Although cognitive performance did not differ, the imaging findings suggest that changes associated with higher FSH may be detectable before clinical symptoms become apparent.

FSH may also influence brain health indirectly through its effects on metabolism. Changes in adiposity, lipid metabolism, and cholesterol homeostasis have all been linked to cognitive decline and Alzheimer’s disease [113,205]. Likewise, vascular dysfunction, dyslipidemia, and chronic low-grade inflammation are well-established features of both metabolic disease and neurodegeneration [113,205]. These observations suggest that any effects of FSH on the brain may extend beyond the central nervous system itself.

##### FSH and Genetic Interactions in Neurodegenerative Processes

The apolipoprotein E (*APOE*) ɛ4 allele, a genetic variant that encodes the ApoE4 protein isoform, increases the risk of neurocognitive impairment, especially in women [215]. Compared with women who are homozygous for the *APOE* ε3 allele, women with one and two copies of the *APOE* ε4 allele have been reported to have approximately 4- and 15-fold higher risks of developing AD, respectively [216]. Mechanistically, animal research shows that ApoE4 and FSH act additively to activate C/EBPβ/δ-secretase signaling, thereby contributing to AD-related pathogenesis [216]. These findings were supported by a study involving 884 postmenopausal women, in which Wang et al. found that FSH levels and *APOE* ε4 carrier status, but not estradiol, significantly and independently affected cerebral β-amyloid deposition [202]. Future research may identify genetic risk patterns that increase vulnerability to neurodegenerative disease in peri- and postmenopausal women.

##### Summary

The idea that FSH may play a role in brain aging represents a shift in how we think about menopause and the brain. For many years, declining estrogen has been the primary explanation for changes in cognition, mood, and neurodegenerative risk. The more recent FSH literature does not challenge the importance of estrogen, but it suggests that the endocrine changes occurring during menopause may be more complex than previously appreciated.

The discovery of FSHRs in brain regions involved in learning and memory, together with experimental studies linking FSH to neuroinflammation, amyloid and tau pathology, and synaptic function, has opened new areas of investigation. Human studies have also reported associations between higher FSH levels and imaging markers of brain aging, including amyloid deposition, white matter changes, and reduced gray matter volume. At the same time, the clinical evidence remains limited, and it remains unclear whether FSH contributes directly to these changes or merely accompanies them. Whether FSH ultimately proves to be a regulator of brain aging, a biomarker of these changes, or both will require further study.

### 4.6. Kidneys

Li et al. [217] examined the relationship between circulating FSH and renal function in a cross-sectional study of 3285 premenopausal, perimenopausal, and postmenopausal women. As expected, FSH increased across the menopausal transition, with median concentrations rising from 8.65 IU/L in premenopausal women to 56.49 IU/L during perimenopause and 71.50 IU/L after menopause. Higher FSH concentrations were associated with poorer renal function among postmenopausal women. Women in the higher FSH quartiles had higher serum creatinine concentrations and lower estimated glomerular filtration rate (eGFR) values (*p* for trend < 0.001). These relationships remained after adjustment for potential confounders. Compared with women in the lowest FSH quartile, those in the highest quartile had approximately twice the odds of reduced eGFR (OR = 2.19, 95% CI: 1.63–2.92) and substantially higher odds of chronic kidney disease (OR = 10.09, 95% CI: 2.28–44.65). The latter estimate, however, should be interpreted with caution given the small number of events and the wide confidence interval.

#### Summary

The kidney has not been studied as extensively as bone, adipose tissue, or the brain. The limited evidence available suggests that higher FSH concentrations are associated with lower eGFR and a greater risk of chronic kidney disease in postmenopausal women. Whether FSH contributes to these changes or simply reflects the broader endocrine changes that occur with aging remains unknown. As with many of the tissues discussed in this review, the kidney represents another area where additional clinical and mechanistic studies are needed.

### 4.7. Immune System

Immunosenescence reflects a gradual age-related deterioration of the immune system and is characterized by changes in immune tolerance, immune cell numbers and function, and chronic inflammation that may result from increased pro-inflammatory cytokine production [218]. The immune system undergoes notable changes during menopause, influencing risk for infectious, chronic, and autoimmune disease (AID) [218].

AID exhibits significant sex differences, with approximately 80% of autoimmune cases occurring in females, suggesting a role for hormones in immune regulation [219]. This hormonal factor is echoed in the relationship between AIDs and reproductive aging. Earlier onset of menopause is observed in several AIDs, including inflammatory bowel disease (IBD), rheumatoid arthritis (RA), systemic lupus erythematosus (SLE), and type 1 diabetes mellitus [220,221]. Concurrently, women with premature ovarian insufficiency (POI) are shown to be 2.6 times more likely to have at least one AID compared to controls [222]. These findings suggest a bidirectional relationship in which ovarian changes may shape immune activity, and immune activation may accelerate ovarian aging.

Much of the evidence surrounding the potential influence of FSH on autoimmune processes pertains to SLE and RA, both of which are associated with higher FSH levels [223,224]. Mechanistically, research suggests that FSH may enhance the expression of pro-inflammatory cytokines, including TNF-α [223]. Though TNF-α is important for normal immune function, excessive production is implicated in some AIDs, including RA, SLE, and IBD [225,226].

Given the rise in immune-mediated conditions among midlife women and its apparent influence on immune and inflammatory signaling, FSH may contribute to the shifting immune landscape of the menopausal transition. Menopause-associated declines in bone mineral density have been partly attributed to immune-mediated mechanisms, which may involve FSH signaling pathways [227].

#### 4.7.1. Preclinical Research and Mechanisms

##### Cellular Mechanisms: FSH Signaling in the Immune System

TNF-α is a pro-inflammatory cytokine primarily produced by immune cells, including macrophages, T lymphocytes (T cells), and natural killer cells [225]. TNF-α is considered a master regulator of the immune response and can stimulate the release of other pro-inflammatory cytokines. Additionally, it may contribute to premature T-cell senescence, potentially impairing immune protection [228,229].

TNF-α exhibits what may be the most direct relationship with FSH, though it appears to be bidirectional. TNF-α contributes to impaired folliculogenesis and ovarian decline by inhibiting FSH-stimulated granulosa cell proliferation, which may consequently suppress estrogen synthesis and FSHR expression [230]. This may contribute to gonadotropin resistance, thus increasing FSH levels.

At the same time, FSH may amplify the inflammatory response, including increased TNF-α levels [227]. Because estrogen suppresses TNF-α production, rising FSH levels and decreasing estradiol levels in perimenopause may have compounding effects on inflammatory and immune signaling [227].

In addition, human cell research suggests that FSH may upregulate macrophage colony-stimulating factor (MCSF) receptors, stimulating macrophage proliferation and cytokine production in the ovaries in the absence of estrogen [231]. This suggests that persistent elevations in FSH may partially explain increased cytokine production by macrophages in aging ovaries.

##### Animal Studies

In a mouse model, Iqbal et al. [227] determined that FSHβ- and estrogen-deficient mice exhibit lower TNF-α levels than controls. Because estrogen suppresses TNF-α production, the authors concluded that FSH appears to directly stimulate TNF-α production from immune cells in bone marrow. Rising FSH levels and decreasing estradiol levels in perimenopause may have compounding effects on inflammatory and immune signaling [227].

In a mouse model of osteoarthritis, Belenska-Todorova et al. [232] determined that FSH increased the percentage of monocytoid CD14+/RANK+ and B cell CD19+/RANK+ cells in bone marrow cultures. This suggests that FSH may accelerate osteoarthritic processes through immune-mediated mechanisms [232].

In another animal model, lipopolysaccharide (LPS) exposure has been associated with transiently elevated FSH levels and decreased FSHR expression, suggesting a potential role for FSH in gut-mediated immune responses affecting the HPG axis [233].

#### 4.7.2. Clinical and Epidemiological Evidence

##### FSH and Immune Dysregulation in Midlife

FSH levels in postmenopausal women have been shown to exhibit a positive correlation with T regulatory (Treg) cells, which would typically suggest a positive effect on immune tolerance [234]. However, simultaneous increased proportions of Th1, Th17, and natural killer cells suggest that Treg cells no longer suppress effector T cell populations, reflecting a potential failed compensatory mechanism in response to a proinflammatory environment [234].

In a cross-sectional study of 69 healthy women (ages 45–60 years), high FSH significantly predicted higher plasma TNF-α levels [228]. Results also demonstrated higher counts of exhausted and senescent T-cells in postmenopausal women compared to premenopausal women, contributing to loss of immune function [228].

##### FSH and Systemic Lupus Erythematosus (SLE)

Emerging research suggests an association between SLE and FSH. A systematic review determined that women with adult-onset SLE exhibited significantly higher FSH levels (*p* < 0.01) compared to healthy controls [235]. However, this study used FSH as a measure of ovarian reserve rather than as a factor in disease activity.

In research comparing postmenopausal women with AID to those without, Ali et al. [224] demonstrated that those with SLE had significantly higher FSH levels compared to controls, in addition to increased levels of erythrocyte sedimentation rate [236] and Immunoglobulin E (IgE), which may be used as markers of disease activity [224,237,238].

##### FSH and Rheumatoid Arthritis (RA)

RA disease activity is shown to initiate or worsen when FSH increases, such as in menopause [223]. In a prospective analysis, female RA patients (mean age 57 years) exhibited significantly higher serum FSH (*p* = 0.025) levels compared to healthy controls, which remained significant after adjusting for age and estradiol levels [223]. Further, FSH in this study group was positively correlated with disease activity when patients were stratified by FSH quartiles. The authors concluded that high FSH may be an independent risk factor for rheumatoid arthritis.

In the aforementioned study by Ali et al. [224], postmenopausal patients with RA exhibited significantly higher FSH levels compared to controls, in addition to increased levels of ESR and IgE, with ESR being more relevant to RA disease activity [224,239].

In a small case–control study involving patients with rheumatoid arthritis, FSH was positively correlated with changes in proinflammatory cytokines, including TNF-α (*p* = 0.001) [58]. Changes in FSH also positively correlated with levels of disease activity markers, including C-reactive protein and ESR, and patient-reported pain levels [58].

##### Is FSH a Therapeutic Target for AIDs?

Though there is an apparent relationship between FSH levels and AID development and activity, causality has not been determined, nor has FSH been established as a therapeutic target in AID. Despite HRT’s suppressive effects on FSH levels, its impact on AID is mixed. HRT may be efficacious in postmenopausal patients with RA and does not appear to contribute to disease flares [240]. Conversely, research suggests an association between HRT and a greater risk of SLE and systemic sclerosis [241].

### 4.8. Stem Cells

FSH is increasingly being recognized as a regulator of more than ovarian follicle development. Emerging evidence suggests that it may also influence stem and progenitor cell populations involved in tissue maintenance and repair [242]. Most of this work has come from reproductive biology, where investigators have begun exploring whether FSH participates in regenerative processes. These findings may have additional implications for menopause.

As this field has expanded, researchers have also started examining whether similar mechanisms extend to non-reproductive tissues [69,243]. Multiple studies have identified FSHR expression on tissue-resident stem and progenitor cells in the ovary, uterus, and bone marrow, suggesting that these cells may respond directly to FSH stimulation [242]. The effects of FSH on stem cells are not uniform and vary according to hormone exposure, tissue type, and timing [244]. Although this area remains under active investigation, FSH levels during menopause may influence regenerative function in multiple tissues.

#### 4.8.1. Preclinical Research and Mechanisms

##### Cellular Mechanisms: FSH Signaling in Stem and Progenitor Cells

In ovarian tissue, FSH has been shown to stimulate self-renewal and clonal expansion of stem and progenitor cell populations expressing FSHRs [245]. Proposed mechanisms include increased proliferation and differentiation following FSHR activation [245,246]. Comparable effects have been observed in uterine and bone marrow-derived stem and progenitor cells, prompting some investigators to propose that FSH directly influences regenerative cell populations [242].

Not all studies point in the same direction. In human endometrial stem cells, exposure to FSH reduced self-renewal, migration, and multilineage differentiation through the PI3K/Akt and ERK1/2 signaling pathways, ultimately impairing endometrial regeneration [244]. These results contrast with reports of FSH-induced stem cell proliferation in other tissues and indicate that the biological effects of FSH depend on tissue type, timing, and hormone exposure levels [244].

FSH interacts with additional regenerative signaling pathways. In an avian ovarian model, FSH acted synergistically with stem cell factor (SCF) to promote primordial follicle assembly, stimulate cell proliferation, activate MAPK and AKT signaling, and reduce apoptosis [247]. The results suggest that FSH functions within a more extensive network of growth factors involved in tissue development and regeneration.

Preclinical research has also implicated FSHR signaling in ovarian cancer development. The FSHR has been identified in stem cells within the ovarian surface epithelium, the tissue from which approximately 90% of ovarian cancers arise [242,248].

Experimental studies further suggest that signaling through the FSHR3 receptor variant may promote ovarian cancer cell proliferation [249,250]. These receptor variants may help explain why studies evaluating FSH and ovarian cancer risk have produced inconsistent findings. Accordingly, FSHR-targeted therapies are now being explored as a potential treatment strategy [251,252].

##### FSH, Stem Cells, and Immune Modulation

FSH may affect the immunomodulatory functions of stem cells. In decidual mesenchymal stem cells, FSH signaling reduced IL-6 production and altered immune regulation pathways [253]. Given that mesenchymal stem cells contribute to tissue repair and immune tolerance, this evidence suggests that FSH may concurrently modulate regenerative and inflammatory processes [253].

Mesenchymal stem cells also serve as precursors to osteoblasts [254]. Experimental evidence suggests that FSH stimulates their proliferation through FSHR-mediated signaling, providing another potential link between FSH, regenerative biology, and bone remodeling [254].

##### FSH and Neural Stem Cells

Beyond reproductive tissues, a recent experimental study demonstrated that FSH enhanced the differentiation of neural stem and progenitor cells and increased the generation of dopaminergic neurons [255]. Although preliminary, these data indicate that FSH-responsive stem cells may have possible applications in treating neurodegenerative diseases.

##### Animal Studies

In mammalian and avian models, FSH exposure has been linked to activation of ovarian stem and progenitor cells, follicle assembly, and germline precursor populations [245,247]. In testicular models, FSH promoted Sertoli cell proliferation and increased glial cell-derived neurotrophic factor (GDNF) production [256]. Viewed together, these findings support the idea that FSH helps shape the regenerative microenvironment of reproductive tissues. Of interest, studies using mesenchymal stem cell treatment in animal models of premature ovarian failure (POF) have reported decreased FSH levels, potentially by restoring ovarian function [257,258].

It is also possible that FSH acts on dental pulp stem cells (DPSC). An animal model using ovariectomized rats demonstrated that FSH negatively regulated mineralization in DPSCs and reduced reparative dentin formation via the JNK signaling pathway [259]. This is relevant to women in menopause because the reduced reparative capacity of the dentin–pulp complex can affect the efficacy of dental procedures (e.g., direct pulp capping).

#### 4.8.2. Clinical Research

Increasing interest in preserving ovarian function has spurred substantial advances in stem cell-based therapies. In preclinical models, mesenchymal stem cells have been shown to enhance follicular survival, ovarian vascularization, hormone production, and tissue repair [260,261]. Early clinical studies have reported partial recovery of menstrual function and hormonal parameters following stem cell-based interventions, although the available evidence remains limited and heterogeneous [260,262].

Interestingly, many of these regenerative strategies intersect with pathways regulated by FSH. Because elevated FSH is a defining feature of both perimenopause and premature ovarian insufficiency (POI), investigators have begun to ask whether changes in stem cell biology contribute to ovarian aging and whether stem cell therapies can modify this process [261,262].

In a small phase I clinical trial, nine women (ages 20–39 years) with unexplained POF and FSH > 25 IU/L received adipose-derived stromal cells (ADSC) transplanted into the ovary [263]. Of the nine women enrolled, four resumed menstruation for several cycles, and four had FSH levels below 25 IU/L. Two patients achieved FSH below 6 IU/L at 12 months [263]. More recently, a meta-analysis found reduced FSH levels after stem cell transplantation in women with POF, though the authors noted high heterogeneity among the studies [264].

ADSCs have also been investigated for their use in treating sexual dysfunction. In one study, women (aged 40 years to menopause) with FSH ≥ 10 IU/L received intravenous ADSCs. Though sexual satisfaction improved in all participants, no significant changes in hormones, including FSH and estradiol, were reported [265].

In a case report of a woman with POF, treatment with bone marrow-derived mesenchymal stem cells (BMSCs) injected into one ovary resulted in an increase in ovary volume in the weeks immediately following injection. At 6 months, her FSH decreased from 89.4 IU/L to 68.5 IU/L, but it increased to 80.3 IU/L at 12 months. She also experienced increased estradiol levels and reduced menopausal symptoms, which were maintained through the end of the 12-month study [266].

Another noteworthy area of clinical relevance is ovarian cancer. Nearly 90% of women diagnosed with ovarian cancer are 45 years and older, coinciding with rising FSH levels [267]. As previously mentioned, FSH signaling through FSHR3 on stem cells within ovarian surface epithelial cells has been proposed as a potential contributor to disease development [242,248].

Results have been historically mixed [268,269]. Recent research found significantly higher FSH levels in patients with epithelial ovarian cancer compared with healthy controls (58.9 vs. 45.5 IU/L, *p* = 0.02) [270]. The highest FSH levels (>72.6 IU/L) with menopause duration >10 years demonstrated a significant association with increased ovarian cancer risk (OR = 5.9, CI = 1.33–26.66, *p* trend = 0.04) [270]. A phase I dose-escalation trial (NCT05316129) is currently underway, evaluating FSHR-targeted immunotherapy in women with FSHR+ epithelial ovarian cancer [271].

#### 4.8.3. Is FSH a Regenerative Signal in Perimenopause?

In summary, these studies suggest that FSH may influence stem and progenitor cell populations involved in tissue maintenance, repair, and regeneration, as well as cancerous cell proliferation. The biological effects of FSH vary depending on tissue type, physiological context, and duration of exposure. While some experimental models show increased proliferation and regenerative signaling, others report impaired stem cell function following prolonged FSH stimulation [242,244].

Rising FSH levels during perimenopause may represent more than a biomarker of declining ovarian reserve. These elevations may also reflect or contribute to tissue remodeling, immune regulation, and regenerative potential associated with reproductive aging. Whether these mechanisms considerably influence menopausal symptoms or long-term health is still unknown, but they present an important area of future research.

## 5. Towards a New Clinical Framework of FSH

Collectively, these findings point towards important clinical questions that have already been discussed to some extent in the scientific literature: (1) Could FSH serve as an early biochemical marker of (reproductive) aging or endocrine dysregulation [94]? (2) Could FSH provide an early indication of the menopausal transition [90]?

While FSH by itself is not a reliable diagnostic marker because its concentration fluctuates throughout the menstrual cycle and is influenced by the timing of measurement, it may still be important to recognize in a clinical setting as more than just a fertility marker. In fact, this variability may itself be purposeful as a pattern-recognition indicator of the ongoing, dynamic signaling that characterizes the HPO axis. Thus, when evaluated longitudinally and interpreted alongside estradiol and other clinical measures, including symptoms, bone mineral density, cardiovascular health, and immune-related changes, FSH could be an essential biomarker that provides preclinical insight into early changes within a woman’s systems biology.

### 5.1. FSH as a Clinical Biomarker

For decades, FSH has been used primarily to assess reproductive status, ovarian reserve, fertility potential, and progression through the menopausal transition. However, as our understanding of FSH biology has expanded, an important question has emerged: Are we fully appreciating the information that this hormone may provide?

#### 5.1.1. Laboratory Interpretation and Reference Ranges

FSH is typically assessed during the early follicular phase (cycle days 2–5), preferably on day 3 in ovulating women. At this time of the menstrual cycle, estradiol and progesterone levels are low, and pituitary gonadotropin output is least affected by cyclical feedback [272,273,274]. During the late reproductive stage, estradiol levels may remain normal or intermittently elevated, which can suppress FSH and obscure underlying changes in pituitary output [89]. This is the reason why FSH is best interpreted in conjunction with estradiol and evaluated over time rather than at a single time point.

Reference ranges for FSH vary by laboratory and assay methodology [275,276] (Table 1). Reported values for FSH are typically expressed in IU/L (or mIU/mL), though absolute values may vary slightly depending on assay calibration and reference standards. In women, FSH concentrations fluctuate across the menstrual cycle and reproductive lifespan, with representative values as follows: follicular phase, approximately 1.4 to 14.6 IU/L; mid-cycle, 4.7 to 23.2 IU/L; luteal phase, approximately 1 to 9 IU/L; and postmenopause, approximately 16 to 157 IU/L. These values will largely depend on the assay and the population studied [277,278,279]. In comparison, reference values in adult men generally range from approximately 1.2 to 15.8 IU/L [278,279]. Thus, postmenopausal women can have up to ten times greater levels than adult men. These ranges highlight the magnitude of the menopausal rise in FSH. It may also suggest greater significance and warrant more clinical attention in women.

Some functional and/or integrative medicine guidelines may propose slightly different, and sometimes narrower interpretive ranges, particularly for early follicular phase testing (e.g., ~2.5 to 10 IU/L) and postmenopause (e.g., ~23 to 116 IU/L). It is worth noting that these values are not universally standardized or validated in large population studies. They should be considered alongside conventional laboratory data [280].

All hormones, including FSH, should be interpreted within a larger physiological and clinical context rather than as a fixed point in time [281]. Although this review aims to foster more clinical discussion of FSH use, it is important to emphasize that there are limitations in determining optimal values for the different stages of menopause [282,283]. As a result, rather than functioning as a specific value at a point in time, FSH may be a better biomarker for assessing the health of the endocrine (HPO) axis over time.

Furthermore, ethnic differences have also been observed in the SWAN cohort [284], where African American women exhibit higher FSH levels than Caucasian women. Estradiol concentrations do not fully explain these differences. In contrast, Chinese and Japanese women have been reported to have FSH levels comparable to those of Caucasian women despite lower estradiol levels. These disparate findings across populations suggest potential differences in endocrine regulation and HPO feedback dynamics. This information may help explain why certain populations experience fewer or more menopausal symptoms than others. It may also provide information about the timing of interventions.

#### 5.1.2. Longitudinal Monitoring

Longitudinal studies have demonstrated that postmenopausal women do not all follow the same endocrine trajectory. In a longitudinal cohort of 291 postmenopausal women from the WHI OsteoPerio study who were not using hormone therapy, investigators characterized FSH patterns over approximately 20 years using group-based trajectory modeling [285]. Three distinct FSH trajectories were identified. The low trajectory (36.1%) showed relatively stable FSH concentrations over time. The moderate trajectory (52.9%) showed increasing FSH levels that extended into the later postmenopausal years. The high trajectory (11.0%) showed initially elevated concentrations, followed by a decline and a later secondary rise.

Women in the moderate and high trajectory groups were more likely to be never smokers, had lower adiposity measures, and reported more severe hot flashes than those in the low trajectory group. These findings suggest that postmenopausal FSH patterns are not homogeneous. They may, in fact, reflect underlying clinical differences in metabolic status and symptom burden, further supporting the idea that FSH can serve as an indicator of endocrine health beyond the final menstrual period.

**Clinical notes**

- FSH should be interpreted together with estradiol and evaluated longitudinally rather than at a single time point, since values fluctuate across the menstrual cycle and during perimenopause.
- Postmenopausal women may have FSH concentrations that are up to ten times higher than those of adult men, highlighting the extent of FSH levels during this time of life.

*Reference ranges vary by laboratory, assay methodology, and population studied.*

#### 5.1.3. Urinary FSH Assessment

A relatively recent development has been the emergence of urine-based FSH testing, primarily for fertility testing [286,287,288]. Up until this point, FSH has been measured through serum assays obtained during the early follicular phase. However, advances in home monitoring technologies now allow women to assess urinary FSH repeatedly over time using immunoassay-based platforms. These approaches have been used primarily for fertility awareness and reproductive tracking, but they also create opportunities for longitudinal monitoring of endocrine aging.

Support for this approach comes from longitudinal studies of urinary FSH dynamics across reproductive aging. In the FREEDOM study, 103 women aged 30–58 years provided 36,786 urine samples over 6–18 months. Investigators observed a progressive increase in early follicular FSH concentrations as women moved through reproductive aging stages, with average values progressing from less than 5 IU/L in women with regular cycles to 5–10 IU/L and eventually greater than 10 IU/L as endocrine patterns approached the menopausal transition [289]. These findings highlight the dynamic nature of FSH and support the value of repeated measurements over time.

Unlike a single laboratory value obtained during an office visit, serial urinary measurements may provide a more complete picture of endocrine dynamics. This information may be particularly useful during perimenopause when hormonal variability is often significant. It could inform clinicians in identifying transitions between reproductive stages, monitoring individual endocrine trajectories, and evaluating responses to therapeutic interventions. While additional validation studies are needed, particularly for applications beyond fertility monitoring, these technologies reflect a much-needed shift toward continuous physiological monitoring and individualized endocrine assessment. As interest in precision medicine and personalized approaches to menopause continues to grow, urinary FSH monitoring may offer a practical tool for tracking reproductive and endocrine aging over time. Data are needed to confirm that urinary FSH levels closely track serum levels.

#### 5.1.4. FSH as a Bone Marker

Due to the preponderance of data on FSH levels relative to bone health, FSH could serve to identify women at risk of accelerated bone loss in perimenopause. Clinically, it may help flag earlier lumbar spine risk, whereas estradiol may be more informative for femoral neck risk [141,142,157]. This signifies FSH’s potential as an early indicator of lumbar spine bone loss and its possible limitations at other sites.

The considerable variability in FSH concentrations across individuals and physiological states complicates its use in screening for low BMD. Considering the fluctuations of FSH during perimenopause, serial evaluation may better inform care than a single FSH measurement. However, Shieh et al. [157] reported that single FSH measurements are likely adequate in identifying women at risk of bone density decline, noting that only 57% and 6% of visits in the SWAN study occurred during the early follicular phase for women in early and late perimenopause, respectively. This suggests that a single measurement may be practical for screening when repeated testing is not feasible.

Several studies have sought to define more specific FSH thresholds for identifying women at risk of bone loss, though balancing sensitivity and specificity is challenging, as lower FSH thresholds are highly sensitive but lack specificity [157]. Sowers et al. [142] suggest that BMD declines significantly starting 2–3 years before the final menstrual period, when FSH levels exceed 34 IU/L. Yet, after conducting a cross-sectional study in women undergoing the menopausal transition, Ramírez Stieben et al. [141] suggested an FSH breakpoint at ~15 IU/L, below which lumbar spine BMD declines rapidly, with a plateau at higher FSH levels. On the other hand, FSH thresholds of ≥16 IU/L in earlier stages and ≥32 IU/L in later stages of perimenopause exhibit distinct sensitivity–specificity profiles for lumbar spine bone loss, indicating that stage-specific thresholds may be more appropriate than a single universal cutoff [157]. In postmenopausal women, an FSH threshold of 49 IU/L is suggested as moderately accurate for detecting low BMD, though estradiol may be better suited for screening in this population [163].

All aforementioned values are considerably lower than the normal upper postmenopausal FSH range of ~157 IU/L [278]. However, a modestly elevated FSH level may serve as an early indicator to initiate proactive care. Proactive care may include further assessment of bone-loss risk factors, early BMD scans, dietary changes, the inclusion of botanicals that support bone health, or the initiation of HRT, as indicated. Importantly, low-weight women (BMI < 20 kg/m^2^) are at increased risk of BMD decline in menopause but may have FSH levels below the diagnostic threshold for menopause (<25 IU/L) [290,291,292]. This point stresses the importance of assessing FSH levels alongside other risk factors for bone loss.

#### 5.1.5. Genetic Testing for FSHR

In addition to circulating FSH levels, genetic association studies have identified links between common variants in the FSH β-subunit gene (*FSHB*) and the *FSHR* gene and features of the female reproductive lifespan [110]. In an analysis of UK Biobank data, including 124,336 women for reproductive lifespan and 36,478 women for menstrual cycle length, variation in *FSHB* (rs11031006) and *FSHR* (rs6166) was associated with differences in menstrual cycle length and reproductive lifespan, suggesting that genetic variation in FSH signaling pathways is associated with interindividual variability in reproductive timing.

As data on receptor variation continues to accumulate, this type of genetic information may provide additional context for navigating the menopausal transition. Much like other gene variants, it may help explain individual differences in reproductive timing and endocrine patterns and support a more personalized, systems-based approach to understanding menopause. At present, genetic testing for *FSHR* variants is not part of routine clinical evaluation of reproductive aging or menopause.

Of note, genetic variation in these same pathways also influences male reproductive physiology, with variants in *FSHB* and *FSHR* associated with differences in serum FSH levels, testicular function, and spermatogenesis, thereby contributing to variability in male fertility parameters [293]. Therefore, both circulating hormone levels and genetic variation may ultimately provide additional insight into the regulation of reproductive aging in both sexes. Despite considerable research over the past two decades, the clinical significance of *FSHR* polymorphisms remains unclear. Studies have reported inconsistent findings across populations, and the underlying mechanisms remain poorly understood. Although variations in both the *FSHR* and *FSHB* genes may collectively influence reproductive traits, the available evidence is not yet strong enough to support their use in clinical practice, particularly because these genetic effects appear to weaken with age.

#### 5.1.6. Pattern Recognition and Precision Medicine

In this era of monitoring metrics and developing algorithms, it is conceivable that a portfolio of clinical markers could be used as a more comprehensive approach to reproductive aging. It might vary by life stage. In perimenopause, FSH can be considered alongside other hormones, such as estradiol, progesterone, testosterone, cortisol, and inhibin B, along with age, ethnicity, lifestyle, nutrition, toxic load, menstrual cycle patterns, and symptoms, in keeping with established staging systems such as STRAW+10 [28] (Table 2). Looking at these factors together provides a more complete picture of the overall pattern and biochemical trajectory of the transition from the reproductive years into early perimenopause. It may also offer insight into any root causes of moderate-to-severe symptomatology.

At the same time, researchers are investigating additional biomarkers of reproductive aging, including circulating microRNAs, menstrual blood markers, epigenetic signatures, ovarian biomechanical properties, and vaginal microbiome profiles. In the future, these emerging tools may help refine the assessment of ovarian aging [294]. This evolving view of reproductive aging and endocrine health takes clinicians beyond a single biomarker, helping them see the whole pattern to better capture endocrine variability and the systemic changes that accompany midlife.

### 5.2. Clinical Interventions to Modulate FSH

To our knowledge, there has not been a broad review of the clinical interventions that affect FSH. Still, studies suggest that FSH responds to a variety of influences, including hormone therapy, diet and specific nutrients, botanicals, environmental exposures, and lifestyle factors. In this section, we summarize the available evidence for each area and consider whether changes in FSH might shape endocrine changes during menopause and inform clinical care.

In current practice, FSH is not usually treated as a therapeutic target. Rather, interventions such as estrogen therapy lower FSH indirectly by restoring feedback within the hypothalamic–pituitary axis. As a result, FSH has generally been viewed as a marker of reproductive and endocrine status rather than as an endpoint in itself.

Hormonal therapies can directly suppress FSH, whereas non-pharmacologic approaches are more likely to affect it through changes in metabolism, environmental exposures, and endocrine signaling. The following sections review what is currently known about the effects of these interventions on FSH in menopausal women.

#### 5.2.1. Hormonal Approaches

Although the WHI [30] remains one of the pivotal studies to influence recommendations of menopausal hormone therapy, FSH was not routinely measured as part of its core protocols. The study was designed to assess clinical outcomes, including cardiovascular disease, fracture risk, and cancer incidence. Sex hormones, including estradiol, were evaluated in select substudies. Therefore, the WHI cannot provide information on the degree, timing, or variability of FSH suppression in response to hormone therapy [30]. For that reason, this review relies on smaller interventional and observational studies in peri- and postmenopausal women that included FSH measurements. Although they are more limited in size, these studies offer a better perspective on how FSH can be altered by hormone therapy.

#### 5.2.2. Exogenous Hormone Therapy and FSH Levels in Peri- and Post-Menopausal Women

##### Oral

Multiple interventional studies demonstrate that FSH is highly responsive to exogenous estrogen therapy in a dose-, time-, and exposure-dependent manner. An early study by Geola et al. [295] evaluated 21 postmenopausal women treated for 6 weeks with oral conjugated equine estrogens (CEE) at 0.15, 0.30, 0.625, or 1.25 mg/day. Dose-dependent reductions in FSH were observed. Baseline FSH concentrations were elevated in postmenopausal women (reported as 2337 ± 213 ng/mL using assay-specific units) compared to premenopausal controls (195 ± 12 ng/mL). Levels declined to 1314 ± 176 ng/mL at the highest dose, representing an approximate 44% reduction (*p* < 0.001). Significant suppression was observed at relatively low doses (≥0.30 mg/day), although FSH levels remained above the premenopausal range even at higher doses. This suggests that CEE was unable to fully normalize hypothalamic–pituitary feedback.

Subsequent randomized data further support a dose-dependent relationship. Yasui et al. [296] evaluated 68 postmenopausal or bilaterally ovariectomized women treated with oral CEE (0.625 mg) plus medroxyprogesterone acetate for 12 months. They found that FSH suppression closely tracked with estradiol concentrations. Reductions in FSH ranged from −22.0% to −41.9% across estradiol strata (*p* < 0.01), with greater sensitivity observed at low-to-moderate estrogen exposures.

Time-dependent effects are also evident. A pharmacokinetic study by Sipinen et al. [297] demonstrated that FSH levels were lower after 1 month of estradiol therapy than after acute administration on day 1. These findings suggest that the suppression of FSH reflects cumulative rather than acute estrogen exposure. Consistent with this finding, Freedman & Blacker [298] reported that 1 mg/day oral 17β-estradiol administered to 12 symptomatic postmenopausal women for 90 days reduced FSH from 58.8 ± 8.9 to 40.1 ± 7.6 IU/L (−31.8%) in the estradiol-treated group.

Short-term data further highlight FSH’s rapid responsiveness. In a study of 168 postmenopausal women, Ushiroyama et al. [299] showed that administration of 2 mg estriol over approximately 2–8 weeks resulted in significant reductions in FSH (*p* < 0.001). Decreases of 39.1–52.2% were noted in patients with effective or markedly effective responses, compared to only 8.2–13.9% in those with limited benefit. These changes occurred without measurable increases in circulating estradiol. This is an interesting finding as it suggests that estriol-mediated suppression of FSH occurs independently of changes in estradiol levels. The magnitude of FSH reduction also correlated with clinical efficacy and side effects, reinforcing its role as a biomarker of endocrine response.

Other data underscore the specificity of this effect to pharmacologic estrogen therapy. In a randomized controlled trial of 60 postmenopausal women, Carmignani et al. [300] demonstrated that continuous combined oral hormone therapy (1 mg estradiol + 0.5 mg norethisterone acetate) significantly suppressed FSH by −46.2% over 16 weeks, whereas soy isoflavones (90 mg/day) increased FSH by +9.0% and placebo by +3.3% (both not significant), despite improvements in menopausal symptoms.

Overall, these findings demonstrate that FSH suppression with oral hormone therapy is rapid, dose-dependent, and sustained. Reductions typically range from ~20% to >50%, depending on treatment intensity and duration. FSH tracks closely with estrogen exposure and clinical response. This reinforces its role as a sensitive indicator of endocrine feedback and systemic endocrine adaptation, rather than merely a marker of ovarian reserve.

##### Oral vs. Transdermal

Oral and transdermal estrogen therapies exhibit similar effects on FSH suppression. Both routes result in dose-dependent, sustained reductions. Chetkowski et al. [301] evaluated 23 postmenopausal women receiving transdermal estradiol (25–200 µg/24 h) or oral CEE (0.625–1.25 mg/day). Both therapies significantly reduced FSH, with a clear dose-dependent decline observed as estradiol exposure increased. At the highest transdermal dose, FSH was reduced by approximately 50% from baseline, whereas oral CEE produced a similarly large suppression at commonly used clinical doses.

Bellantoni et al. [302] studied 31 healthy postmenopausal women (28 completers) undergoing four sequential 8-week treatment periods with escalating doses of transdermal 17β-estradiol (0, 50, 100, and 150 µg/day) in a double-blind design. FSH declined progressively from a pooled baseline of 132 ± 17 IU/L to 57 ± 4 IU/L, 41 ± 4 IU/L, and 34 ± 4 IU/L across increasing doses, corresponding to approximate reductions of ~57%, ~69%, and ~74%, respectively. Co-administration of medroxyprogesterone acetate further reduced FSH at each dose level (*p* < 0.01–0.02), indicating an additive suppressive effect of the progestin on hypothalamic–pituitary signaling.

Observational data further support comparable effects across routes. In a cohort of 117 women receiving oral or transdermal HRT, Kawakita et al. [303] reported significant reductions in FSH at both 6 months and 1 year (*p* < 0.001). Median FSH declined from 88.5 IU/L at baseline to 49.0 IU/L at 6 months and 45.2 IU/L at 1 year, corresponding to approximate reductions of ~45% with oral estrogen and ~38% with transdermal estrogen. There was no significant difference between routes (*p* = 0.162).

Long-term randomized data from the Kronos Early Estrogen Prevention Study [304] reinforce these findings. Over 48 months, both oral CEE and transdermal 17β-estradiol produced sustained reductions in FSH, with decreases of ~19–30% and 24–38%, respectively, depending on the time since menopause.

Overall, studies show that both oral and transdermal estrogen therapies lower FSH to a similar extent. Although the two routes differ in how estrogen is absorbed and metabolized, changes in FSH seem to depend more on overall estrogen exposure than on the method of delivery.

##### Vaginal

In a randomized, placebo-controlled trial, Fernandes et al. [305] assigned postmenopausal women to vaginal estrogen (*n* = 18), vaginal testosterone (*n* = 19), or placebo lubricant (*n* = 20) over 12 weeks to assess potential systemic endocrine effects. FSH levels showed no statistically significant changes within or between groups. In the estrogen group, mean FSH levels were 78.4 ± 31.1 IU/L at baseline, 84.6 ± 28.6 at 6 weeks, and 73.9 ± 23.2 at 12 weeks (between-group *p* = 0.51). In the testosterone group, values were 77.5 ± 32.5, 78.8 ± 29.9, and 76.4 ± 32.5 (*p* = 0.94), whereas in the placebo group, values were 71.3 ± 24.5, 70.1 ± 23.2, and 74.4 ± 25.5 (*p* = 0.63). These findings suggest that, at the doses studied, locally administered vaginal hormones have little effect on circulating FSH and limited impact on systemic endocrine feedback.

##### Systematic Reviews/Meta-Analyses

A systematic review and meta-analysis of 21 studies (*n* = 1285 women) [171] found that HRT lowers FSH. Both oral and transdermal estrogen therapies were associated with significant reductions in FSH compared to pre-treatment levels (oral: SMD −1.89; transdermal: SMD −1.21; *p* < 0.00001 for both), whereas vaginal estrogen had no significant effect. The degree of FSH reduction did not differ significantly between routes of administration or across HRT regimens. Decreases were evident within 3–6 months and persisted with longer treatment, suggesting an early and sustained effect of estrogen on the hypothalamic–pituitary axis. These findings are consistent with a 2025 systematic review and meta-analysis [306], which found that HRT led to significantly greater reductions in FSH compared to placebo in controlled trials [SMD −0.65 (−1.05, −0.24), *p* = 0.002], despite heterogeneity across studies (I^2^ = 72%, *p* = 0.01).

##### HRT–FSH–BMD Axis

In a retrospective study of 125 women on HRT (43 perimenopausal and 82 postmenopausal women), Kawai et al. [173] monitored longitudinal changes in sex hormones and BMD over 24 months. Subjects received either oral CEE (0.625 mg daily or cyclic), transdermal 17β-estradiol patches (72 μg every other day), with medroxyprogesterone acetate administered in women with an intact uterus.

Estradiol increased significantly from 39.3 ± 76.6 pg/mL at baseline to 71.0 ± 67.9 pg/mL at 12 months and remained elevated at 68.3 ± 54.5 pg/mL at 24 months (*p* < 0.01 vs. baseline). In parallel, FSH decreased significantly from 67.9 ± 36.3 IU/L to 47.9 ± 29.0 IU/L at 12 months and further to 45.3 ± 24.4 IU/L at 24 months (*p* < 0.01), which corresponds to a reduction of approximately −29.5% at 12 months and −33.3% at 24 months from baseline across mixed HRT regimens. Lumbar spine BMD improved significantly during the first 12 months (0.933 ± 0.157 to 0.938 ± 0.152 g/cm^2^; *p* < 0.05), with no further significant gains at 24 months (0.936 ± 0.150 g/cm^2^). Changes in estradiol were positively associated with changes in BMD, whereas higher FSH was associated with lower BMD. These findings suggest that changes in FSH parallel changes in skeletal health during treatment.

##### Clinical Significance and Summary from HRT Findings

Hormone therapy consistently lowers FSH in peri- and postmenopausal women. Mechanistically, this occurs by restoring the estradiol-mediated feedback at the hypothalamic–pituitary axis. Oral and transdermal preparations produce similar reductions in FSH, whereas low-dose vaginal estrogen has little effect, likely due to its limited systemic absorption.

The degree of FSH suppression depends on estrogen dose. Most changes occur during the first 3–6 months of treatment before a plateau is reached. Combined estrogen-progestin regimens may further reduce FSH, although this effect has not been consistently observed or studied. A common question is whether hormone therapy can return FSH to premenopausal levels. However, this is not typically observed. Therefore, when it comes to clinical intervention, it may be less important to normalize to a value and more about modulating in response to symptoms and disease risk.

#### 5.2.3. Diet, Nutrient, and Botanical Interventions

Dietary, nutrient, and botanical interventions may alter FSH through any number of indirect effects on metabolic, endocrine, and receptor-level signaling pathways. In contrast to pharmacologic therapies, which directly suppress FSH, these approaches tend to produce changes that reflect shifts in nutrient status and hormonal feedback. Unfortunately, to date, there is a paucity of human clinical data specifically evaluating the effects of diet and nutrition on FSH modulation, particularly in peri- and postmenopausal populations. Based on limited evidence, this section explores how data on dietary patterns and plant-based compounds may influence FSH and their potential role in shaping endocrine function.

#### 5.2.4. Dietary Patterns and Foods

##### Probiotics

In a randomized, controlled study of perimenopausal women, Szydłowska et al. [307] evaluated the effects of a multi-strain probiotic formulation containing *Lactobacillus* and *Bifidobacterium* species administered over a 12-week period. At the end of the study, FSH increased significantly from 31.91 to 42.00 IU/L (+ 31.6%, *p* < 0.009) after probiotic intake. These findings suggest that modulation of the gut microbiota may influence HPO axis signaling; however, in this study, the intervention was associated with an increase rather than a suppression of FSH. Given the gut’s important role in menopause, extensive research exploring different probiotic strains and their effects on neuroendocrine markers such as FSH would be of interest [308].

##### Soy and Wheat Flour Interventions

In a randomized, double-blind trial of postmenopausal women [309], participants consumed approximately 45 g/day of soy or wheat flour for 12 weeks following a baseline observation period. Both interventions reduced vasomotor symptoms, but only the wheat flour group demonstrated a statistically significant reduction in serum FSH, decreasing from 82.93 ± 3.85 to 74.84 ± 4.64 IU/L over 12 weeks (−9.8%, *p* < 0.05). No significant change was observed in the soy group (84.52 ± 4.63 to 83.17 ± 4.62 IU/L). There was no significant difference between groups (*p* = 0.32). These findings suggest that changes in FSH are not directly linked to phytoestrogen exposure. It is also important to note that some individuals may not tolerate wheat due to gluten sensitivity or celiac disease, which could limit the applicability of wheat-based interventions in certain populations.

#### 5.2.5. Nutrients

##### Melatonin

In a randomized, placebo-controlled study of perimenopausal and menopausal shift-working women (*n* = 22), low-dose melatonin (0.3 mg nightly for three months) significantly improved sleep quality on days off and reduced climacteric symptoms without altering reproductive hormone levels, including FSH [310]. Changes in FSH were highly variable and not significantly different between intervention and placebo groups across shift conditions: morning shift (−30.7 ± 100.4% vs. 144.1 ± 91.4%), afternoon shift (29.3 ± 139.0% vs. 41.7 ± 97.0%), and night shift (92.9 ± 101.8% vs. −28.1 ± 95.0%), with no significant group or shift effects (*p* = 0.791 and *p* = 0.962, respectively). These findings indicate that physiological-dose melatonin may improve symptoms and circadian alignment without directly affecting FSH.

In contrast, higher-dose interventions appear to engage endocrine pathways more directly. In a clinical study of perimenopausal women with sleep disorders (*n* = 15 for hormone analysis), melatonin supplementation (3 mg nightly, administered 30 min before bedtime for 3 months) was associated with significant improvements in sleep quality and changes in reproductive hormones [311]. Specifically, melatonin was associated with a significant reduction in FSH (*p* < 0.05), with parallel changes in LH and estradiol. This finding would suggest modulation of the HPG axis rather than suppression of FSH alone. However, absolute hormone values and percentage changes were not reported, limiting the understanding of the extent of FSH reduction.

Similarly, in a randomized, placebo-controlled trial of women aged 42–62 years, melatonin supplementation (3 mg nightly for six months) was associated with reductions in FSH in women with low baseline melatonin levels [312]. A negative correlation was observed at baseline between FSH and endogenous melatonin levels. Following six months of treatment, a reduction in FSH was observed in melatonin-treated women with low baseline melatonin levels.

##### Clinical Summary: Melatonin and FSH

Based on available data, melatonin does not appear to be a reliable or primary intervention for lowering FSH in peri- or postmenopausal women. At physiological doses (~0.3 mg), it consistently improves sleep and circadian symptoms without meaningful changes in FSH. At higher doses (e.g., 3 mg), modest reductions in FSH may occur, but these effects are inconsistent, subgroup-dependent (e.g., low baseline melatonin, sleep disruption), and not well quantified.

Overall, these findings suggest that melatonin does not uniformly suppress FSH but may modulate its expression in different contexts. At physiological doses, melatonin primarily improves circadian alignment and symptom burden without significantly altering gonadotropins, whereas at higher doses or in individuals with reduced endogenous melatonin, it may influence FSH dynamics.

##### Soy Isoflavones

Across randomized, placebo-controlled trials in peri- and postmenopausal women, soy isoflavones show variable, generally modest effects on circulating FSH. Outcomes are influenced by dose, duration, and physiological context. In an 8-week double-blind trial of postmenopausal women (*n* = 61; 30 intervention, 31 control), daily intake of ~54 mg isoflavones via soy biscuits resulted in a statistically significant reduction in FSH, decreasing from 76.93 ± 19.37 to 62.24 ± 18.14 IU/L (−19.1%, *p* = 0.001). However, there was also a concurrent decline in the placebo group, from 78.99 ± 23.42 to 68.45 ± 19.22 IU/L (−12.6%, *p* = 0.001). Therefore, only a moderate net treatment effect was observed when comparing both groups [313].

In contrast, multiple trials report no significant treatment-specific effects. A 12-week randomized trial providing 70 mg/day of isoflavones to postmenopausal women with elevated baseline FSH (>40 IU/L) found no significant between-group differences, despite improvements in other metabolic and hormonal markers [314]. Similarly, in a randomized controlled trial of 60 postmenopausal women, isoflavone supplementation (90 mg/day, *n* = 20) over 16 weeks did not suppress FSH. A modest increase of +9.0% was observed [300]. In a randomized crossover trial of 40 postmenopausal women, short-term supplementation with soy protein (~44 mg/day isoflavones), with or without probiotics, did not significantly affect FSH compared with a milk protein control. There was no differential effect of soy or probiotics on reproductive hormones [315].

Context-dependent effects are seen in select studies. In a 6-month trial of 160 perimenopausal women, reductions in FSH were observed only when soy isoflavones were combined with calcium, whereas isoflavones alone did not significantly alter FSH [316]. Specifically, FSH increased in the placebo (*n* = 37), isoflavone (*n* = 38), and calcium (*n* = 38) groups, while the combined calcium plus isoflavone group (*n* = 37) showed significant reductions (−13.7 ± 5.7 and −8.7 ± 3.6 IU/L at 3 and 6 months, respectively; *p* < 0.05 vs. all groups), suggesting a synergistic rather than independent effect. A systematic review and meta-analysis with 47 studies reported significant reductions in FSH in premenopausal women (SMD, *p* = 0.01), no statistically significant effects in postmenopausal populations, and limited data in perimenopause [317]. Similarly, a more recent large-scale meta-analysis of 40 randomized controlled trials in postmenopausal women found no significant effect of soy isoflavones on FSH, estradiol, or other measures of estrogenicity [318].

##### Clinical Summary: Soy Isoflavones and FSH

The effects of soy isoflavones on FSH in peri- and postmenopausal women appear to be modest and inconsistent. Although some studies have reported reductions in FSH, these changes are often small, and in some cases, similar declines are also seen in the placebo groups. Most randomized trials in postmenopausal women, including studies using doses of approximately 50–90 mg/day, have found little or no effect on circulating FSH.

The response also appears to depend on the clinical context. In one study, FSH declined only when isoflavones were combined with calcium, suggesting that any benefit may result from combination approaches rather than soy alone. Consistent with these findings, several meta-analyses have reported no significant effect of soy isoflavones on FSH or other markers of estrogen activity in postmenopausal women.

Overall, soy isoflavones do not appear to be a reliable strategy for lowering FSH. While they may exert mild estrogen-like effects, their influence on hypothalamic–pituitary signaling is variable and likely depends on factors such as menopausal stage and concurrent interventions.

##### Vitamin D

Vitamin D has been associated with variation in FSH levels across the menopausal transition. In a 2-year prospective study of 100 women aged 45–55 years, vitamin D deficiency (<20 ng/mL) was associated with significantly higher FSH levels compared with sufficient levels (~69.8 vs. ~33.6 IU/L, *p* < 0.01), as well as lower estradiol concentrations [319]. Consistent with these findings, a cross-sectional study of 92 women (46 premenopausal and 46 postmenopausal), aged 20–65 years and recruited from a fertility center, reported significantly higher FSH and lower vitamin D levels in postmenopausal than in premenopausal women. Both parameters were associated with advancing age [320].

##### Clinical Summary: Vitamin D and FSH

Assessing vitamin D status may add useful context when interpreting FSH levels during the menopausal transition. Lower 25-hydroxyvitamin D (25(OH)D) concentrations have been associated with higher FSH and lower estradiol levels. These findings suggest that changes in FSH during menopause reflect not only ovarian aging but also other factors, such as nutrient status. In peri- and early postmenopausal women, evaluating 25(OH)D alongside FSH may help identify potentially modifiable contributors to hormonal patterns and symptoms. Correcting vitamin D deficiency could be part of a systems-biology clinical approach. However, studies directly examining whether vitamin D influences FSH signaling, or whether the two act together to affect menopausal health, are still lacking. These are intriguing findings that prompt inquiry into other vitamin levels during the menopausal transition.

#### 5.2.6. Botanicals (Listed Alphabetically)

##### *Asparagus racemosus* (Shatavari) Root Extract

In a randomized, double-blind, placebo-controlled trial, Yadav et al. [321] investigated the effects of a standardized *Asparagus racemosus* (shatavari) root extract (200 mg/day) in 49 perimenopausal women with mild-to-moderate climacteric symptoms. After 120 days, women receiving shatavari experienced a 56.3% reduction in FSH levels (64.23 ± 6.05 to 28.04 ± 3.15 U/L, *p* < 0.001), whereas the placebo group showed a slight increase of 3.74%. Because symptom improvement was the primary outcome and hormone levels were secondary measures, it is not possible to determine whether the observed clinical benefits were directly related to changes in FSH.

Additional mechanistic clues come from an in silico study showing that several bioactive compounds in *Asparagus racemosus*, including shatavarins, can bind to the FSHR [322]. While these findings suggest that shatavari may influence FSH signaling, computational studies alone cannot determine whether these interactions alter circulating FSH levels or clinical outcomes.

##### *Cimicifuga racemosa* (Black Cohosh)

Across multiple clinical studies, *Cimicifuga racemosa* has shown no effect on FSH, despite improvements in menopausal symptoms. In an 8-week study, treatment with an ethanolic extract (Remifemin^®^) lowered LH levels without altering FSH levels, suggesting selective effects on hypothalamic–pituitary signaling [323]. Similarly, randomized trials in breast cancer survivors and postmenopausal women found no significant changes in FSH after treatment with *C. racemosa* extracts, despite symptomatic benefit [324,325].

Thus, the available evidence suggests that *C. racemosa* does not meaningfully influence FSH regulation. Its effects on menopausal symptoms therefore appear to occur through mechanisms other than gonadotropin suppression. From a clinical perspective, *C. racemosa* may offer symptom relief without substantially altering FSH levels, supporting its use as a non-hormonal option during menopause.

##### Combination Formula (Black Cohosh, Soy Isoflavones, Lignans)

In a randomized, double-blind, placebo-controlled trial of a combined botanical formulation (black cohosh, soy isoflavones, and lignans), participants experienced improvements in menopausal symptoms, with only modest reductions in FSH (−6.7%, *p* < 0.01). Therefore, in this instance, with a specific botanical combination, there was an influence on endocrine signaling, but the effects on pituitary gonadotropin output are relatively small [326]. Different doses, combinations, and concentrations of actives may yield different responses. More research is needed to understand how varying levels and types of phytochemicals could affect FSH, if at all.

##### *Dioscorea villosa* (Wild Yam Extract)

In a double-blind, placebo-controlled, crossover trial involving 23 postmenopausal women, topical wild yam (*Dioscorea villosa*) extract administered over 3 months did not produce any statistically significant changes in circulating FSH levels compared with placebo, despite baseline FSH concentrations in the postmenopausal range (~74 IU/L) [327].

##### *Elaeagnus angustifolia* Whole Fruit Powder

In a 10-week randomized, placebo-controlled trial [328] in postmenopausal women, supplementation with *Elaeagnus angustifolia* whole fruit powder (15 g/day) was associated with a within-group increase in FSH from 40.94 to 53.73 IU/L (+31.2%, *p* = 0.020). A similar increase was observed in the placebo group (39.78 to 54.41 IU/L; ~36.8% increase), with no significant between-group difference, which indicates no meaningful effect of the intervention on FSH relative to placebo.

##### Fennel and Evening Primrose Oil

A triple-blind, randomized controlled trial in 125 postmenopausal women evaluated the effects of fennel (a phytoestrogen-rich botanical) and evening primrose oil over 8 weeks [329]. Both interventions resulted in significant reductions in circulating FSH and increases in estradiol compared to placebo (*p* < 0.001), with no significant difference between the two treatment groups (*p* = 0.304). FSH decreased by approximately 23% in the fennel group and 30% in the evening primrose oil group, whereas no meaningful change was observed in the placebo group. Improvements were also observed in psychological menopausal symptoms, while physical and urogenital symptoms were largely unchanged.

##### *Lepidium meyenii* and *Lepidium peruvianum* Chacón

*Lepidium meyenii* has not demonstrated consistent effects on circulating FSH across randomized, placebo-controlled trials in peri- and postmenopausal women. In a crossover trial of 29 postmenopausal Chinese women, Stojanovska et al. [330] reported that FSH concentrations remained stable after 3.3 g/day of maca for 6 weeks per phase, with no significant differences from placebo. Similarly, in a randomized, double-blind, placebo-controlled trial of perimenopausal Japanese women, Takewaka and Hara [331] found that 300 mg/day of a standardized maca extract for 8 weeks improved menopausal symptoms and increased estradiol in women with regular menstrual cycles, but had no significant effects on FSH or other gonadotropins. Consistent with these findings, Brooks et al. [332] demonstrated, in a crossover study of postmenopausal women, that 3.5 g/day of maca for 6 weeks improved psychological symptoms and sexual function without altering FSH, estradiol, LH, or SHBG.

Although three clinical trials have evaluated *Lepidium meyenii* with respect to FSH, only one preparation, Maca-GO^®^, a standardized, pre-gelatinized, concentrated proprietary blend of specific phenotypes of *Lepidium peruvianum* Chacón, has demonstrated statistically significant hormonal effects by modulating the HPO axis. Reductions in FSH were observed following supplementation with *Lepidium peruvianum* Chacón (Maca-GO^®^) across three clinical studies in early postmenopausal women (total *n* = 182). The effect size was dependent on study sequence and comparator [333,334,335]. These studies were conducted primarily in healthy, predominantly Caucasian early postmenopausal women and involved the administration of 2 g/day of a phenotype-specific Maca-GO^®^ preparation for 2 to 8 months.

Due to the unique crossover and multi-sequence designs, percent changes in FSH were calculated using study-specific reference points (including baseline and/or placebo-phase values, depending on sequence allocation and the reported contrast). Using this approach, reductions in FSH after approximately 2 months of supplementation ranged from ~11.4% to 52.1% across study arms. Most values clustered between ~11.4% and 25%, while a smaller subset of comparisons showed larger reductions exceeding 50% in specific sequence arms.

In the Meissner et al. [335] study (*n* = 31), which included both 2- and 4-month intervention periods, reductions in FSH remained within a similar overall range (~12–55%), indicating that the effect was maintained over the extended 4-month duration rather than consistently increasing over time. In a cohort treated for up to 8 months in the same study, FSH levels declined by approximately 27.6% from baseline and by up to 34.2% compared with placebo. FSH remained relatively stable during the placebo phases, which supports the use of placebo-adjusted comparisons to assess treatment effects. Although findings across studies vary due to differences in study design and analysis, overall they suggest that Maca-GO^®^ affects HPO signaling in early postmenopausal women. Further supporting this mechanism, effects on estradiol and progesterone were also observed in some trials with Maca-GO^®^.

A bone density sub-analysis in a small subset of participants from one Maca-GO^®^ trial [335] suggests both skeletal and hormonal changes. Over four months, the placebo group (*n* = 4) showed declines in bone density measures, including total density and Z-score, whereas the Maca-GO^®^ group (*n* = 5) showed increases relative to both baseline and the placebo group. Total bone density increased (379.1 to 388.0 mg/10^3^ mm), and Z-score improved (0.36 to 0.52) with Maca-GO^®^, while both declined in the placebo group. Cortical and subcortical densities followed similar patterns, whereas trabecular density remained relatively stable in both groups. These changes occurred alongside a marked reduction in FSH (approximately 55%) and a more than twofold increase in estradiol (~135%) in the Maca-GO^®^ group. Hormonal changes in the placebo group were minimal.

##### *Nigella sativa* Extract

In a single-blind, placebo-controlled trial involving 50 postmenopausal women [336], *Nigella sativa* extract administered at 910 mg/day and 1365 mg/day for 8 weeks increased estradiol concentrations and improved vaginal maturation index. However, FSH levels remained non-significant relative to baseline (*p* > 0.05), while the placebo group exhibited a significant rise in FSH that was consistent with the natural postmenopausal trajectory.

##### Red Clover

Evidence from both individual trials and pooled analyses suggests that red clover isoflavones have little effect on FSH in peri- and postmenopausal women. In a 12-month randomized, placebo-controlled trial, red clover supplementation did not significantly alter FSH levels compared with placebo [337]. Likewise, a systematic review and meta-analysis found no significant overall effect (pooled SMD −0.812; 95% CI: −1.93 to 0.312; *p* = 0.157), despite substantial variability among studies [338]. Intervention durations ranged from approximately 10 weeks to 12 months, and most commonly around 12 weeks.

Yet not all trials have reported the same results. In one 16-week randomized study, women taking a standardized red clover supplement (40 mg/day) experienced an approximately 18% reduction in FSH (from 59.3 to 48.6 IU/L), whereas FSH increased in the placebo group [339]. Although this study suggests that red clover may lower FSH under certain conditions, the body of literature indicates an inconsistent, generally modest effect across populations.

Overall, the available evidence does not support red clover as a reliable intervention for reducing FSH, and any effects on gonadotropin levels should be interpreted with caution.

##### *Rheum rhaponticum* (ERr 731^®^)

To date, the ERr 731^®^ literature has not systematically evaluated FSH as a clinical or mechanistic endpoint [340]. Its observed effects are thought to be mediated primarily through selective estrogen receptor activity, rather than through direct modulation of hypothalamic–pituitary signaling.

##### *Vitex agnus-castus* (Chaste Tree)

Preclinical studies suggest that the effects of *Vitex agnus-castus* on FSH depend on the underlying hormonal environment. In a diabetic rat model marked by impaired reproductive function, treatment with an ethanolic extract increased FSH, along with LH, estradiol, and progesterone, suggesting recovery of HPG axis activity [341].

In contrast, in a mouse model of premature ovarian failure, Vitex lowered FSH while improving ovarian morphology, increasing follicle number, and enhancing estradiol production [342]. These findings point to a potential normalizing effect of Vitex on gonadotropin regulation rather than a simple increase or decrease in FSH.

These observations come entirely from animal studies. To the best of our knowledge, no studies have examined the effects of *Vitex agnus-castus* on FSH in perimenopausal or postmenopausal women. There are, however, studies exploring its use in women with PMOS and menstrual difficulties, such as premenstrual syndrome [343,344,345]. Specifically, one clinical trial in women with PMOS given 400 mg Vitex and 750 mg myo-inositol did report a positive change in FSH, in addition to other hormonal markers [346].

##### *Withania somnifera* (Ashwagandha) Root Extract

Vani et al. [347] conducted a randomized, double-blind, placebo-controlled trial in 60 perimenopausal women (ages 45–55 years) to evaluate the effects of *Withania somnifera* (ashwagandha) root extract on menopausal symptoms over a 56-day intervention period. Secondary outcomes included changes in reproductive hormones, including FSH. The intervention group demonstrated a statistically significant reduction in circulating FSH concentrations compared with placebo (mean change: −1.00 ± 0.60 IU/L vs. +0.40 ± 6.00 IU/L; −1.6%; *p* < 0.001). There were concomitant increases in estradiol (22.20 to 23.02 pg/mL; mean change +1.00 ± 6.00 pg/mL) and progesterone (0.28 to 0.43 ng/mL; mean change +0.15 ± 0.96 ng/mL). Although these changes reached statistical significance, the overall magnitude was modest.

##### Clinical Summary: Nutritional and Botanical Interventions

Most botanical interventions have little or inconsistent effects on circulating FSH levels, particularly in peri- and postmenopausal women (Table 3). This suggests that many botanicals improve symptoms through mechanisms other than direct suppression of pituitary hormones.

A few botanicals have been associated with reductions in FSH, but the findings vary in both magnitude and reproducibility. In one randomized trial, *Asparagus racemosus* (shatavari) lowered FSH by 56.3% over 120 days in perimenopausal women, one of the largest effects reported to date; however, this was a small study and would need replication. Fennel and evening primrose oil have shown more moderate reductions of approximately 23% and 30%, respectively. Results for *Lepidium meyenii* indicate that it does not tend to affect FSH. However, the standardized Maca-GO^®^ formulation has been associated with reductions ranging from approximately 11% to 52%, with larger effects reported in some subgroups.

By contrast, several commonly used botanicals, including *Cimicifuga racemosa* (black cohosh), red clover, *Nigella sativa*, and *Dioscorea villosa* (wild yam), have shown little or no effect on FSH despite improvements in menopausal symptoms. Combination formulas may produce small reductions, but these effects are generally modest. Animal studies suggest that *Vitex agnus-castus* may help normalize FSH depending on the underlying endocrine state, although this has not been confirmed in menopausal women. *Withania somnifera* (ashwagandha) has shown statistically significant but relatively small effects.

Overall, except in select cases, substantial suppression of FSH by botanicals appears uncommon. When changes are observed, they tend to be preparation-specific, modest in magnitude, and influenced by the underlying hormonal environment. 

#### 5.2.7. Lifestyle Factors

##### Environmental Toxicants: Endocrine Disrupting Chemicals (EDCs) and Heavy Metals

A systematic review of endocrine-disrupting chemicals (EDCs) found that exposure to these compounds is consistently associated with changes in FSH and other reproductive hormones [351]. In a cross-sectional analysis of 612 U.S. women from NHANES 2017–2018, higher serum concentrations of several per- and polyfluoroalkyl substances (PFAS), including n-PFOA, PFOS, PFNA, and PFHxS, were associated with higher FSH and lower estradiol and progesterone levels [352]. Each interquartile range increase in log-transformed PFNA was associated with a 42.0% increase in ln-FSH (*p* = 0.01). Even though associations were stronger in premenopausal women, these findings remain relevant to peri- and postmenopausal populations.

Similar findings have been reported in other PFAS studies. In a case–control study of 371 women, higher concentrations of novel PFAS compounds were associated with elevated FSH and lower markers of ovarian reserve [353]. In contrast, an analysis of glyphosate exposure in NHANES data from 2017 to 2018 found reductions in sex steroids but no significant associations with FSH or LH after adjustment for potential confounders [354].

Heavy metals have also been linked to altered FSH levels. In women aged 42–60 years, higher cadmium exposure was associated with higher FSH concentrations, independent of BMI [355]. In perimenopausal women, higher urinary cadmium was significantly correlated with increased FSH across BMI strata. Both increased cadmium burden and higher FSH were independently associated with lower femoral BMD [355]. In postmenopausal women with lower BMI, incremental increases in FSH were linked to a higher likelihood of osteoporosis (OR 2.78; 95% CI: 1.43–5.42). Likewise, a cross-sectional analysis of 3798 women found that higher blood lead levels were associated with greater FSH [356]. Elevated FSH was inversely associated with total femur BMD, and, in postmenopausal women, lower lumbar spine density and greater fracture risk [356].

Overall, these findings suggest that environmental exposures, including PFAS, cadmium, and lead, may influence FSH dynamics in midlife and older women. They also show that FSH may be a promising biomarker linking environmental, endocrine, and skeletal health.

##### Smoking

Current smoking was associated with significantly higher circulating FSH levels in a cross-sectional study of 188 reproductive-age women (ages 22–49 years) [357]. Differences were observed across age groups, with levels approximately 6.9 IU/L higher at age 45 than in nonsmokers of the same age. Yet smoking was not associated with antral follicle count, inhibin B, or estradiol. No associations were observed between FSH and alcohol or caffeine intake. However, these findings are derived from reproductive-age populations, so it remains unclear how smoking-related elevations in FSH translate to perimenopausal and postmenopausal physiology.

There may be a connection between smoking and cadmium in the observed effects on FSH, as cigarettes are a major non-dietary source of cadmium exposure [358]. This point raises the possibility that tobacco-related cadmium burden could influence FSH levels. Thus, from a clinical perspective, it may be worthwhile to assess FSH in smokers or in those with increased cadmium burden.

##### Physical Activity

Xin et al. [350] conducted a randomized controlled trial in 52 perimenopausal women (aged 45–55 years) to evaluate the effects of Tai Chi Rouli Ball exercise on bone mineral density and bone metabolism markers. The authors reported reductions in circulating FSH; however, the presented data indicate that FSH levels increased in both the exercise group (31.5 → 35.4 IU/L; +12.4%) and the control group (30.3 → 32.3 IU/L; +6.6%) over the intervention period. A greater relative increase was observed in the exercise group.

In a randomized, controlled trial, Jorge et al. [349] evaluated the effects of a 12-week Hatha yoga or exercise intervention in 88 postmenopausal women. These activities were performed twice weekly, compared with a control group that did not perform them. Menopausal symptoms and hormonal parameters were assessed. The yoga intervention consisted of a structured session including asanas (postures, 45 min), pranayama (breathing exercises, 10 min), relaxation (10 min), and meditation (10 min). The exercise intervention included stretching routines targeting the shoulder girdle, cervical musculature, and lower extremities, similar to protocols used in labor gymnastics, performed in seated or standing positions. The control group underwent a 12-week waiting period without intervention. FSH was measured at baseline and post-intervention. Baseline levels were similar across groups (control: 50.26 IU/L; exercise: 43.24 IU/L; yoga: 41.43 IU/L). After 12 weeks, the control group exhibited an increase in FSH to 64.98 IU/L (+29.3%), consistent with the expected postmenopausal trajectory. In contrast, the exercise group demonstrated a reduction in FSH to 37.90 IU/L (−12.3%) while the yoga group remained relatively stable at 42.50 IU/L (+2.6%). Between-group differences were statistically significant (*p* = 0.001).

These findings suggest that structured physical activity may attenuate the expected postmenopausal rise in FSH, whereas yoga may help stabilize gonadotropin levels. However, the extent of change in FSH levels remains modest.

##### Resistance Training

Nilsson et al. [348] conducted a randomized controlled trial in 65 postmenopausal women with vasomotor symptoms to evaluate the effects of a 15-week resistance training intervention on FSH levels. In participants completing an average of two training sessions per week, there was a modest reduction in FSH that did not reach statistical significance (−3.5 ± 16.3 IU/L vs. +3.2 ± 16.2 IU/L; *p* = 0.063). In a responder analysis of women achieving a ≥50% reduction in hot flush frequency, FSH did not change significantly. Finally, no association was observed between changes in FSH and improvements in vasomotor symptoms over the 15-week period.

##### Acupuncture

Wang et al. [359] and Sunay et al. [360] provide evidence from randomized, sham-controlled trials indicating that acupuncture-based interventions do not significantly modulate circulating FSH levels in postmenopausal women, despite symptom improvement. In a study of 72 early postmenopausal women, Wang et al. [348] demonstrated that 6 weeks of electroacupuncture, with a 12-week follow-up, did not result in significant changes in FSH compared with sham treatment. No meaningful within- or between-group differences were observed. Similarly, Sunay et al. [360], in a trial of 53 postmenopausal women, reported significant improvements in menopausal symptoms with acupuncture relative to sham, yet found no statistically significant differences in FSH between groups. Overall, these findings suggest that the symptomatic benefits of acupuncture in menopause may occur independently of measurable changes in FSH concentrations.

##### Clinical Summary: Lifestyle Interventions

FSH appears to respond to some lifestyle factors, though studies to date suggest these effects are generally inconsistent and modest. Importantly, the limited influence of lifestyle interventions on FSH does not diminish their potential value during menopause. Many of these behaviors may still offer meaningful benefits to overall health and well-being, regardless of whether they substantially alter FSH levels.

Most promising is the association between environmental exposures to EDCs such as PFAS, cadmium, and lead and elevated circulating FSH levels, accompanied by reductions in ovarian hormones. These patterns are observed across midlife and postmenopausal populations and extend to skeletal outcomes: higher FSH is linked to lower BMD and increased fracture risk. In contrast, it has been shown that glyphosate may reduce sex steroid levels without a corresponding increase in FSH. Smoking is also associated with elevated FSH, with higher levels observed in women approaching midlife, independent of changes in estradiol or ovarian reserve markers. This effect may reflect altered hypothalamic–pituitary signaling or toxicant-related effects, including those associated with cadmium exposure. Despite the differing effects of various EDCs, it remains essential to consider the total toxic load throughout the menopausal trajectory to help preserve endocrine health.

Physical activity shows variable and generally modest effects. Some structured exercise interventions may attenuate the expected postmenopausal rise in FSH or produce small reductions in FSH. Others may increase FSH despite reported benefits. Yoga-based interventions appear to stabilize FSH relative to untreated progression. Overall, the activities tested in these studies resulted in only a relatively small change in FSH. Even so, regular movement remains an important part of healthy aging and menopausal care, regardless of its effect on FSH levels. The type, intensity, and timing of exercise may also need to be individualized to best support endocrine health.

Finally, two studies using acupuncture as an intervention demonstrate a consistent dissociation between symptom improvement and FSH dynamics, although more studies are needed. Randomized controlled trials show no significant changes in FSH despite improvements in menopausal symptoms.

In summary, lifestyle factors appear to influence FSH indirectly through broader effects on environmental exposures, metabolism, and neuroendocrine regulation. In peri- and postmenopausal women, FSH may reflect the combined impact of these physiologic inputs rather than serve as a primary target of lifestyle interventions. At the same time, additional research is needed to better understand how different lifestyle strategies affect women during midlife and the menopausal transition.

## 6. Conclusions

Since its discovery, FSH has been viewed primarily through the lens of reproduction. Over time, its roles in follicular development, steroidogenesis, and the menopausal transition have become well established. This foundational framework remains fundamental to our understanding of endocrine physiology and has served its purpose. Yet the evidence reviewed herein would suggest that FSH may indicate more than just ovarian aging (Table 4).

Several observations challenge a purely reproductive view of FSH. The discussion begins with the fact that FSH is present in both women and men, even though its name implies a function limited to ovarian follicles. There are, then, more recent findings from the 21st century that help clarify its name and function. First, FSH rises early during reproductive aging, often years before sustained declines in estradiol become apparent. Second, FSH-responsive receptors have been identified in multiple tissues beyond the gonads, including bone, adipose tissue, immune cells, vascular tissues, and the brain. Third, experimental and clinical studies increasingly associate elevated FSH with physiological changes that occur during the menopausal transition and extend beyond fertility or ovarian function alone. These observations do not negate the traditional model; they suggest that it may represent only one layer of a more complex physiological reality, and that FSH may be better described as a Polyendocrine Synchronization Hormone.

Such a dynamic, shifting perspective aligns with the growing appreciation for systems biology and network physiology. Biological systems rarely operate through isolated signaling pathways. Rather, they function through intricate networks in which hormones, immune mediators, metabolites, neural inputs, and cellular signaling pathways continuously interact. The endocrine system itself has undergone a conceptual expansion over the past several decades, evolving from a circuit of isolated glands to a more comprehensive communication network that includes adipose tissue, bone, skeletal muscle, the gastrointestinal tract, and the central nervous system. Thus, it becomes increasingly difficult to view reproductive aging as a process confined solely to the ovary or to estradiol loss alone.

Menopause offers a useful example of this complexity. The transition is accompanied not only by changes in reproductive function but also by alterations in bone remodeling, body composition, metabolic regulation, vascular physiology, immune activity, cellular aging, and brain health. Historically, these changes have been largely attributed to declining estrogen levels. While estrogen undoubtedly plays a central role, the timing and breadth of these physiological changes suggest that additional endocrine signals may also contribute. The marked rise in FSH, occurring precisely at the life stage when many of these changes emerge, suggests that FSH participates in this larger, network-like adaptive process.

Experimental, epidemiologic, and clinical studies increasingly link FSH to changes in bone, adipose tissue, metabolism, vascular biology, neuroendocrine function, immune modulation, and even stem cell physiology. Even though the strength of the evidence differs across these organ systems, the overarching observation is the same (Table 5). The sum of these findings supports the idea that FSH participates in the physiological adaptations that accompany midlife and menopause. Therefore, in addition to its well-established role as a marker of declining ovarian reserve, the long-term sequential trajectory of FSH over a woman’s reproductive years and into mid-life may act as a potential indicator of changes in interactions among multiple endocrine networks. Furthermore, it might even be implied that FSH could signal aging processes across systems biology networks.

The intervention studies reviewed in this manuscript further reinforce this perspective and provide a critical distinction. FSH is highly responsive to endocrine manipulation through pharmacological means, but it is not uniformly or directly modifiable across intervention types. Systemic hormone therapy remains the most consistent modulator of FSH with rapid, dose-dependent, and sustained reductions of 20–70% depending on exposure and duration. In contrast, nutritional, botanical, environmental, and lifestyle interventions generally produce smaller and more variable effects. For the majority, their effects are typically indirect and may involve metabolic, receptor-level, or neuroendocrine pathways. This is demonstrated in targeted interventions with specific botanical preparations where measurable reductions in FSH can occur in select populations, but no effect or even increases in others. On the whole, there are many factors to consider, such as botanical preparation and dose, baseline physiology and reproductive stage of the women studied, and study design.

Also, it must be acknowledged that there is a paucity of significant data to make strong conclusions about the role of FSH in menopause. Overall, current findings suggest that FSH is highly responsive to endocrine state, yet unlikely to be a therapeutic target in its own right. Instead, it may be most useful as a surrogate, upstream, pituitary-based biomarker that reflects the cumulative effects of endocrine, metabolic, and environmental influences. As such, the available data do not support positioning FSH as a singular driver of aging or disease. Many mechanistic questions remain unresolved, and evidence for extragonadal actions is stronger in some tissues than in others. For example, bone has the strongest association with FSH, whereas evidence linking FSH to adipose tissue and metabolic physiology is less robust. Findings in neuroendocrine, immune, vascular, and stem cell biology remain preliminary and require further study. Recognizing these differences in the strength of the evidence will be important as the field moves toward clinical translation. The goal is not to replace or dismiss the traditional reproductive framework of FSH, but to build upon it while remaining grounded in the available science. Future studies that include FSH as a predefined endpoint, particularly in relation to skeletal, metabolic, neuroendocrine, immune, and aging outcomes, will be essential for clarifying the extent of its impact and its physiological and clinical significance.

To summarize, the question of whether follicle-stimulating hormone is misnamed cannot be answered definitively at this time based on available science. The name remains historically accurate and reflects the biological activity by which the hormone was first discovered. However, while it may be an accurate term, the prevailing evidence suggests that follicle-stimulating hormone may no longer fully capture the breadth of FSH biology, as presented in this review.

In a similar way, the recent shift from the term polycystic ovarian syndrome to polyendocrine metabolic ovarian syndrome (PMOS) reflects a growing recognition that endocrine disorders extend beyond reproductive tissues and involve complex interactions across multiple physiological systems [9]. Thus, the nomenclature may lag behind the research findings. The same shift has been observed in the reframing of adipose tissue from a passive storage depot to an active endocrine organ. Therefore, FSH may be undergoing such a transition.

Ultimately, the pressing question may not only be whether follicle-stimulating hormone is misnamed, but also whether its biology has been viewed too narrowly in light of more recent scientific findings. Based on what is known thus far, FSH is more accurately conceptualized as a pituitary-derived synchronizing signal that reflects the whole-body endocrine state. The more important question may not be whether FSH is misnamed, but whether its biology has been viewed too narrowly. As research continues to evolve, FSH may prove to be a more informative marker of women’s health during the menopausal transition than previously appreciated.

## Figures and Tables

**Table 1:** Reference Laboratory Ranges for FSH (IU/L).

Clinical Context	Representative FSH Values (IU/L)	Interpretation
**Follicular phase**	1.4–14.6	Best time for testing in ovulating women (cycle days 2–5, preferably day 3). During this time, pituitary output is least affected by cyclical hormone fluctuations.
**Mid-cycle**	4.7–23.2	Reflects the ovulatory surge and normal menstrual cycling.
**Luteal phase**	1–9	Lower values due to ovarian feedback later in the menstrual cycle.
**Postmenopause**	16–157	Indicates the loss of ovarian feedback and the marked rise in FSH that accompanies menopause.
**Adult men**	1.2–15.8	Supports spermatogenesis and Sertoli cell function.

**Table 2:** Proposed Clinical Framework for the Assessment of FSH in Midlife and Menopause.

Clinical Domain	What to Assess	Clinical Application
**Hormone assessment**	FSH together with estradiol, progesterone, testosterone, cortisol, and inhibin B, if available	Measure FSH during the early follicular phase (days 2–5, ideally day 3) in menstruating women. Interpret trends over time rather than relying on a single value. Higher FSH levels over time may provide an earlier signal of reproductive aging than estradiol alone.
**Menopausal staging**	Age, menstrual cycle patterns, symptoms, and STRAW+10 stage	Consider changes in cycle length and overall symptom burden alongside laboratory values.
**Bone health** **evaluation**	Bone mineral density (DEXA or REMS), fracture risk factors, bone turnover markers, and body weight	Consider earlier screening in women with rising FSH, particularly those with low BMI or other osteoporosis risk factors.
**Cardiometabolic** **assessment**	Lipids, glucose, insulin resistance, body composition, blood pressure, and waist circumference	Interpret FSH within the broader context of metabolic health and adiposity.
**Immune and** **inflammatory status**	Autoimmune history, inflammatory burden, and immune-related symptoms	Use FSH as supportive context rather than a diagnostic tool.
**Genetic factors**	Variants in *FSHR* and *FSHB*	Genetic testing is not currently recommended for routine menopause care, but may eventually help explain differences in endocrine aging trajectories and symptoms.
**Nutritional** **assessment**	Overall dietary pattern, nutritional status, and intake of nutrients relevant to menopause and bone health (e.g., calcium and vitamin D)	Evaluate nutrition within the context of endocrine, skeletal, and metabolic health.
**Botanicals and** **supplements**	Menopausal hormone therapy, nutrients, and botanicals discussed in the review	Individualize interventions according to symptoms, menopausal stage, and risk profile.
**Lifestyle assessment**	Sleep, stress, physical activity, smoking status	Consider lifestyle factors that shape the endocrine milieu during the menopausal transition and may influence symptoms.
**Environmental health**	Exposure to endocrine-disrupting chemicals and other environmental toxicants	Assess environmental exposures as part of a systems-based evaluation of reproductive and endocrine aging.

**Table 3:** Clinical Trial Data on the Effect of Pharmaceutical, Dietary, Nutrient, and Lifestyle Interventions on Serum FSH Levels in Peri- and Post-Menopausal Women. Each section is organized by efficacy, beginning with the most effective therapeutic interventions and proceeding to the least effective.

Therapy	Reference	Intervention	% Change in FSH Levels	Duration	Subjects	Statistical Significance (Yes/No)
Exogenous hormones						
	Bellantoni et al., 1991 [302]	Escalating doses of transdermal estradiol (up to 150 µg/day) with cyclic medroxyprogesterone acetate	~57% to ~74% reduction	8 weeks	28 postmenopausal women	Yes, *p* < 0.0001
	Ushiroyama et al., 2001 [299]	2 mg estriol	39.1 to 52.5% reduction	2–8 weeks	168 postmenopausal women	Yes, *p* < 0.001
	Carmignani et al., 2010 [300]	Continuous combined oral hormone therapy (1 mg estradiol + 0.5 mg norethisterone acetate)	46.2% reduction	16 weeks	20 postmenopausal women	Yes (*p* < 0.01)
	Kawakita et al., 2025 [303]	Oral or transdermal estrogen	~45% and ~38% reductions for oral estrogen and transdermal, respectively	6 months and 1 year	Postmenopausal women; *n* = 76 (oral), *n* = 41 (transdermal)	Yes, *p* < 0.0001
	Geola et al., 1980 [295]	Oral hormone replacement therapy (CEE), different doses	44% reduction at the highest dose of estrogen (1.25 mg/day)	6 weeks	21 postmenopausal women	Yes, *p* < 0.001
	Yasui et al., 2001 [296]	0.625 mg conjugated equine estrogen (CEE, Premarin, Wyeth) and 2.5 mg medroxyprogesterone acetate (MPA, Provera, Upjohn) given either every other day or every day	22 to 41.9% reduction, depending on low, middle, or high levels of circulating estradiol	12 months	68 postmenopausal women	Yes, *p* < 0.01
	Kling et al., 2019 [304]	Oral CEE or transdermal estradiol	~19–30% reduction for oral estrogen and ~24–38% reduction for transdermal estrogen	48 months	Postmenopausal women at different time periods from when they reached menopause; *n* = 109 (oral) and *n* = 107 (transdermal)	Yes, *p* < 0.001
	Freedman & Blacker, 2002 [298]	1 mg/d 17beta-estradiol orally	31.8% reduction	90 days	12 postmenopausal women	Yes, *p* < 0.02
	Kawai et al., 2004 [173]	Oral CEE (0.625 mg daily or cyclic), transdermal 17β-estradiol patches (72 μg every other day), with medroxyprogesterone acetate administered in women with an intact uterus	29.5 to 33.3% reduction	12–24 months	43 perimenopausal women and 82 postmenopausal women	Yes, *p* < 0.01
	Fernandes et al., 2018 [305]	Vaginal estrogen, vaginal testosterone (T), or placebo lubricant	No effect	12 weeks	Postmenopausal women *n* = 18 (vaginal estrogen), *n* = 19 (vaginal T)	No
Diet & Botanicals						
	Yadav et al., 2025 [321]	Standardized *Asparagus racemosus* root extract (shatavari), 200 mg daily	56.30% reduction	120 days	49 perimenopausal women	Yes (*p* < 0.001)
	Meissner et al., 2006 a [335]	2 g Maca-GO^®^ (phenotype-specific maca)	~12–55% reduction	2 months or 4 months	31 Caucasian healthy early postmenopausal women	Not stated
	Meissner et al., 2006 b [334]	2 g Maca-GO^®^ (phenotype-specific maca)	~11–52% reduction	2 months	130 Caucasian healthy early postmenopausal women	Yes, *p* < 0.05
	Ghavi et al., 2023 [329]	Fennel oil, evening primrose oil (EPO)	23% and 30% reductions in the fennel oil and EPO interventions, respectively	2 months	Postmenopausal women; N = 43 (fennel) and 42 (EPO)	Yes, *p* < 0.001 compared with placebo; however, fennel and EPO groups were not statistically different.
	Husain et al., 2015 [313]	~54 mg isoflavones via soy biscuits vs. control (non-specified)	19.1% reduction with soy biscuits and 12.6% reduction with placebo	8 weeks	Postmenopausal women; N = 61; 30 soy biscuits, 31 control	Yes, *p* = 0.001
	Jeri, 2002 [339]	A single daily tablet of Promensil^®^ containing 40 mg of standardized isoflavones (genistein, daidzein, formononetin, and biochanin).	18% reduction	16 weeks	15 healthy postmenopausal women	Yes, *p* < 0.001
	Meissner et al., 2005 [333]	2 g Maca-GO^®^ (phenotype-specific maca)	~15–34% reduction	2 months or 8 months	21 Caucasian healthy early postmenopausal women	Not stated
	Murkies et al., 1995 [309]	45 g/day of soy or wheat flour	9.8% reduction with wheat flour (1.6% in the soy flour group)	12 weeks	Postmenopausal women; N = 28 (soy flour) and N = 30 (wheat flour)	Yes, *p* < 0.05
	Pokushalov et al., 2025 [326]	Combination of black cohosh (25 mg), soy isoflavones (40 mg), and SDG lignans (20 mg)	6.7% reduction	90 days	96 postmenopausal women	Yes, *p* < 0.01
	Vani et al., 2026 [347]	300 mg ashwagandha root extract powder, one capsule twice daily	1.6% decrease	56 days	30 healthy women aged 45–55 years with a clinical diagnosis of menopause	Yes, *p* < 0.001
	Muho, 2025 [319]	Vitamin D levels	FSH levels were ~108% higher in women with vitamin D deficiency compared to those with normal levels.	N/A	100 menopausal women, grouped by Vitamin D levels (< 20 ng/mL and >30 ng/mL)	Yes, *p* < 0.01
	Zhang et al., 2020 [316]	Four groups: control, soy isoflavone, calcium, and soy isoflavone combined with calcium groups	No percentage provided (only absolute values); Only the combined calcium plus isoflavone group (*n* = 37) showed significant reductions (−13.7 ± 5.7 and −8.7 ± 3.6 IU/L at 3 and 6 months, respectively; *p* < 0.05 vs. all groups)	6 months	160 perimenopausal women	Yes, *p* < 0.05 for the soy isoflavone + calcium group only
	Ghazanfarpour et al., 2015 (systematic review and meta-analysis) [338]	Red clover	Limited effect, not specified	10 weeks to 12 months, most commonly around 12 weeks	449 peri- and postmenopausal women experiencing menopausal symptoms, particularly vasomotor symptoms (hot flashes)	No
	Chen et al. 2023 [311]	3 mg melatonin	Stated there was a reduction, but without specific values	3 months	15 perimenopausal women with sleep disorders (hormone analysis done)	Not stated
	Nettleton et al., 2005 [315]	Soy protein (~44 mg/day isoflavones), with or without probiotics providing 10^9^ CFU of *Lactobacillus acidophilus* (DDS™-1) and *Bifidobacterium longum* plus 15–20 mg/day fructooligosaccharides	No effect	Four 42-day diet periods in a randomized, crossover design	Postmenopausal women; N = 40 (N = 20 postmenopausal breast cancer survivors and 20 healthy postmenopausal women)	No
	Kim et al., 2013 [314]	Either 70 mg of isoflavone or placebo capsules daily	No effect	12 weeks	42 Korean postmenopausal women	No
	Stojanovska et al., 2015 [330]	3.3 g/day of maca or placebo (crossover trial)	No effect	6 weeks	29 postmenopausal Hong Kong Chinese women	No
	Takewaka and Hara, 2018 [331]	300 mg Maca-BG1.2^TM^ or 360 mg placebo	No effect	8 weeks	42 healthy peri-menopausal Japanese women	No
	Brooks et al., 2008 [332]	3.5 g/day of powdered maca for 6 weeks and a matching placebo for 6 weeks	No effect	6 weeks for each intervention	14 postmenopausal women	No
	Saraiva et al., 2026 [310]	0.3 mg melatonin	No effect	3 months	22 perimenopausal and menopausal women (nurses doing shift work)	No
	Düker et al., 1991 [323]	*Cimicifuga racemosa* (Remifemin^®^)	No effect	8 weeks	Menopausal women	No
	Nappi et al., 2005 [325]	*Cimicifuga racemosa* (Remifemin^®^), 40 mg daily	No effect	3 months	32 postmenopausal women	No
	Komesaroff et al., 2001 [327]	Wild yam cream	No effect	3 months	23 postmenopausal women	No
	Sukatendal et al., 2025 [336]	*Nigella sativa* seed extract at 910 mg/day and 1365 mg/day	No effect	8 weeks	40 postmenopausal women	No
	Atkinson et al., 2004 [337]	Red clover-derived isoflavone tablet (26 mg biochanin A, 16 mg formononetin, 1 mg genistein and 0.5 mg daidzein)	No effect	12 months	56 postmenopausal women with Wolfe P2 or DY mammographic breast patterns	No
	Dubey et al., 2024 [340]	*Rheum rhaponticum* (ERr 731^®^)	Not evaluated	N/A	N/A	N/A
		*Vitex agnus-castus*	Not evaluated	N/A	N/A	N/A
	Szydłowska et al., 2021 [307]	Sanprobi^®^ Barrier, 2.5 × 10^9^ CFU/day, 3 times daily dosing	31.6% *increase*	5 weeks	25 perimenopausal and postmenopausal women	Yes, *p* = 0.009
	Emaminia et al., 2020 [328]	15 g of *Elaeagnus angustifolia* whole fruit powder	31.2% *increase*	10 weeks	30 postmenopausal women	Yes, *p* = 0.02
	Carmignani et al., 2010 [300]	90 mg soy isoflavones/day	9% *increase*	16 weeks	20 postmenopausal women	Yes, *p* < 0.01
Lifestyle						
	Nilsson et al., 2022 [348]	Resistance training three times per week	5.5% decrease in the intervention group and a 3.8% *increase* in the control group.	15 weeks	33 postmenopausal women with vasomotor symptoms	*p* = 0.063
	Jorge et al., 2016 [349]	Yoga intervention consisted of a structured session including asanas (postures, 45 min), pranayama (breathing exercises, 10 min), relaxation (10 min), and meditation (10 min)	2.6% *increase*	12 weeks	40 postmenopausal women	Yes, *p* < 0.001
	Xin et al., 2024 [350]	Tai Chi Rouli Ball exercise	12.4% *increase*	Three times a week for 6 months. Each session lasted approximately one hour.	27 perimenopausal women (aged 45–55 years)	Yes, *p* < 0.05

**Table 4:** Reframing FSH: From a Reproductive Hormone to a Potential Marker of Systemic Endocrine Aging.

Historical View of FSH	Emerging Perspective
**Primary function:** Regulates ovarian follicle development and estradiol production in women; supports spermatogenesis in men.	**Broader role:** May participate in physiological processes extending beyond reproduction and ovarian function.
**Primary site of action:** Gonads (ovaries and testes).	**Potential sites of action:** Receptors have been identified in bone, adipose tissue, vascular tissues, monocytes, hepatocytes, neuronal tissues, and mesenchymal stem cells.
**Clinical significance:** Used primarily to assess ovarian reserve, reproductive aging, infertility, and menopausal status.	**Potential clinical significance:** May serve as an earlier marker of endocrine aging and provide insight into changes occurring across multiple organ systems during midlife.
**Timing during menopause:** Viewed largely as a compensatory response to ovarian failure and declining estrogen.	**Timing during menopause:** FSH often rises years before sustained declines in estradiol, suggesting that endocrine changes begin earlier than previously recognized.
**Interpretation of menopause:** Menopausal symptoms and age-related changes are attributed primarily to estrogen deficiency.	**Interpretation of menopause:** Menopause may reflect coordinated changes across multiple endocrine tissues, with FSH representing one component of a broader physiological transition.
**Bone:** Bone loss understood primarily because of estrogen deficiency.	**Bone:** The strongest evidence for extragonadal FSH activity exists in bone, where FSH may influence osteoclast differentiation, bone turnover, and fracture risk.
**Body composition and metabolism:** Midlife changes in adiposity and muscle mass are attributed mainly to aging and estrogen withdrawal.	**Body composition and metabolism:** Experimental and clinical studies suggest possible links between FSH, adipose tissue biology, lean mass, lipid metabolism, insulin sensitivity, and mitochondrial function.
**Cardiovascular and brain health:** Considered secondary consequences of reproductive aging.	**Cardiovascular and brain health:** Emerging evidence suggests possible relationships between FSH and vascular remodeling, mood, cognition, and neuroendocrine function, although causality remains uncertain.
**Conceptual framework:** FSH is primarily a reproductive hormone.	**Conceptual framework:** FSH may represent both a reproductive hormone and a marker and perhaps a mediator or signaler of endocrine aging.

**Table 5:** Strength of Evidence Rankings for the Effect of FSH on Body Systems.

Body System (s)	Key Findings Discussed in the Paper	Strength of Evidence
**Bone**	FSH receptors have been identified on osteoclasts and osteoclast precursors. Multiple mechanistic, animal, epidemiologic, and clinical studies link elevated FSH levels with increased bone turnover, lower bone mineral density, and fracture risk during the menopausal transition.	Strong
**Body composition (adipose tissue and skeletal muscle)**	Functional FSH receptors have been identified in adipocytes. Preclinical studies suggest effects on lipid storage, thermogenesis, and energy expenditure. Human studies have associated higher FSH with changes in visceral fat and lean mass, although findings are not entirely consistent.	Moderate
**Metabolism**	Studies have reported associations between FSH and glucose regulation, adipokines, insulin resistance, mitochondrial function, and lipid metabolism. However, these relationships are influenced by adiposity, menopausal stage, and the overall hormonal environment.	Moderate
**Cardiovascular system**	FSH trajectories have been associated with measures of vascular remodeling and cardiometabolic risk, including carotid intima-media thickness and endothelial health. Clinical findings remain mixed and vary across populations and stages of menopause.	Limited to moderate
**Brain and neuroendocrine system**	FSH receptors have been reported in neuronal tissues, and observational studies suggest potential links between FSH and mood, cognition, and neuroendocrine changes during menopause. Mechanistic evidence remains limited.	Limited to moderate
**Immune system and inflammation**	FSH receptors have been identified on monocytes and other immune-related cells. Experimental studies suggest effects on inflammatory signaling pathways, although clinical evidence is still limited.	Limited
**Stem cells and regenerative biology**	FSH receptor expression has been described in mesenchymal stem cells and progenitor populations. Most evidence is preclinical.	Preliminary
**Kidney and renal system**	Observational studies suggest associations between higher FSH levels and less favorable measures of renal function, but mechanistic and longitudinal data are lacking.	Preliminary

## Data Availability

No new data were created or analyzed in this study. Data sharing is not applicable to this article.
